# The white spot syndrome virus wsv156 protein hijacks Parkin-dependent mitophagy to promote viral infection

**DOI:** 10.1128/jvi.00418-26

**Published:** 2026-07-29

**Authors:** Yan Wang, Lu-Lu Yang, Kai-Ming Cai, Xuan Zeng, Ling-Ke Liu, Hai-Peng Liu

**Affiliations:** 1 State Key Laboratory of Marine Environmental Science, State-Province Joint Engineering Laboratory of Marine Bioproducts and Technology, College of Ocean and Earth Sciences, Xiamen University, Fujian Ocean Innovation Center 554979, Xiamen, Fujian, China; 2 Laboratory for Marine Biology and Biotechnology, Pilot National Laboratory for Marine Science and Technology 474988 , Qingdao, China; Wageningen University & Research, Wageningen, Netherlands

**Keywords:** white spot syndrome virus, wsv156, mitochondria, mitophagy, mito-aggresome, Drp1, Parkin, incomplete mitophagy

## Abstract

White spot syndrome virus (WSSV), a major pathogen causing severe losses in global crustacean aquaculture, ranks among the largest known animal enveloped DNA viruses. While some features of WSSV–host interactions are known, the mechanisms by which the virus exploits host mitochondrial functions to promote its replication remain unclear. Here, using the red claw crayfish (*Cherax quadricarinatus*) model, we demonstrate that WSSV infection activates host mitophagy. This process is orchestrated by the mitochondria-targeting viral protein wsv156, which acts as a key effector that triggers the formation of mitochondrial aggregates (mito-aggresomes) and initiates mitophagy. Mechanistically, wsv156 recruits and activates dynamin-related protein 1 (*Cq*Drp1) to drive mitochondrial fission and aggregation. Concurrently, wsv156 facilitates the recruitment of the E3 ubiquitin ligase Parkin (*Cq*Parkin) to mitochondria, engaging the canonical PTEN-induced putative kinase 1 (*Cq*PINK1)–Parkin pathway to initiate mitophagy. Intriguingly, the viral envelope protein VP26 blocks the final step of autophagic degradation, arresting the process in an incomplete state. We further show that this incomplete mitophagy benefits viral replication through a dual mechanism: it enhances glycolysis to meet the heightened energy and biosynthetic demands of infection while suppressing cell apoptosis to preserve cellular viability. Collectively, our findings uncover how a large DNA virus exploits and precisely modulates host mitophagy, specifically by initiating and then arresting it to craft a supportive intracellular niche for replication. This study not only advances the understanding of WSSV pathogenesis but also provides evolutionary insights into conserved viral strategies for manipulating mitochondrial quality control across diverse hosts, from crustaceans to mammals.

**IMPORTANCE:**

White spot syndrome virus (WSSV) poses the most significant viral threat to global crustacean aquaculture. A major barrier to developing effective countermeasures is the limited understanding of its molecular pathogenesis. This study demonstrates that WSSV actively hijacks host mitochondrial quality control by triggering a stalled form of mitophagy. We identify wsv156 as a key orchestrator of this process, driving mito-aggresome formation and activating Parkin-dependent mitophagy while arresting its completion via VP26, then creating a stalled, incomplete state. Importantly, this incomplete mitophagic response is repurposed to benefit viral replication through a dual mechanism: it enhances glycolysis to fuel viral biosynthesis and concurrently suppresses host cell apoptosis to maintain cell viability. Our findings uncover a novel strategy, whereby an enveloped large DNA virus manipulates mitochondrial turnover to create a favorable replicative niche. These results deepen the mechanistic understanding of WSSV infection and highlight wsv156 as a potential target for future antiviral strategies in aquaculture.

## INTRODUCTION

White spot syndrome virus (WSSV), an enveloped DNA virus and the sole member of the genus *Whispovirus* (family *Nimaviridae*), causes substantial economic losses in crustacean aquaculture, particularly in shrimp and crayfish. Its large (~300 kbp) circular double-stranded DNA genome encodes approximately 532 putative open reading frames (ORFs) (1). Early WSSV research focused on structural proteins such as VP28, VP26, VP24, and VP19, which are involved in viral entry and assembly, as well as non-structural proteins like immediate-early protein 1 (IE1), wsv021, and VP9, which regulate viral transcription (2–4). In recent years, attention has shifted toward understanding how WSSV systemically manipulates host organelles. For example, wsv406 localizes to the endoplasmic reticulum and promotes viral replication by modulating the unfolded protein response (5). Growing evidence also indicates that WSSV hijacks host redox homeostasis and aerobic glycolysis to secure metabolic resources for its replication (6, 7). To date, however, only a handful of WSSV ORFs have been shown to interact with host enzymes involved in energy metabolism. Crucially, how WSSV affects mitochondrial fate and function, particularly whether it actively intervenes in key mitochondrial quality control pathways such as mitophagy, remains poorly understood.

Mitochondria are dynamic organelles that serve as central hubs for energy production, redox balance, apoptosis regulation, and innate immune signaling, making them critical targets in virus–host interactions. Their functional integrity is maintained through quality control mechanisms, with mitophagy representing the primary pathway for clearing damaged mitochondria (8). In crustaceans, the role of mitophagy during viral infection remains largely unexplored. Previous studies have shown that WSSV infection induces marked mitochondrial abnormalities, including cristae disorganization and vacuolization (9). Emerging evidence suggests viral involvement in this process: for instance, the mitochondria-localized protein wsv152 promotes apoptosis, though its regulatory mechanisms are not fully defined (10). Additionally, the viral envelope protein VP26 inhibits autophagosome–lysosome fusion, thereby disrupting late stages of autophagic degradation (11). These observations raise a key question: does WSSV actively manipulate host mitochondrial quality control, particularly the mitophagy pathway, to reshape the intracellular environment and facilitate viral replication?

Mitophagy is typically initiated by stress-induced mitochondrial fission, which depends on the GTPase activity of Drp1 (12). Drp1 recruitment drives mitochondrial division and facilitates activation of the PINK1–Parkin pathway. Upon mitochondrial depolarization, stabilized PINK1 recruits the E3 ubiquitin ligase Parkin, leading to ubiquitination of outer membrane proteins such as translocase of outer mitochondrial membrane 20 (TOMM20) and voltage-dependent anion channel (VDAC). These ubiquitin chains then recruit autophagy receptors and microtubule-associated protein 1 light chain 3 (LC3)-labeled membranes, ultimately targeting damaged mitochondria for lysosomal degradation (13). In vertebrates, diverse viruses, including hepatitis B virus (HBV), Epstein-Barr virus (EBV), and influenza A virus (IAV), commonly exploit this pathway. By promoting Drp1-dependent fission and hijacking PINK1–Parkin-mediated mitophagy, viruses can suppress apoptosis, reprogram host metabolism, and evade immune detection, thereby enhancing replication (14). Some viral proteins, such as varicella-zoster virus (VZV) glycoprotein E and SARS-CoV-2 ORF10, even mimic host mitochondrial proteins to directly localize to mitochondria and induce mitophagy (15, 16).

Beyond these shared strategies, certain viruses employ more refined mechanisms to modulate mitophagy progression. For example, the EBV protein BHRF1 triggers Drp1-dependent fission and promotes the formation of mito-aggresomes that are subsequently cleared via mitophagy (17). Other viruses, including human parainfluenza virus type 3 (HPIV3) and SARS-CoV-2, initiate mitophagy but subsequently block autophagosome–lysosome fusion, stalling the process at a late stage (18, 19). Viral manipulation of mitophagy is often coupled with metabolic reprogramming, notably the upregulation of glycolysis in a manner analogous to the Warburg effect. During HBV or Newcastle disease virus (NDV) infection, for instance, mitophagy-mediated stabilization of hypoxia-inducible factor 1α (HIF1α) enhances glycolysis, supplying biosynthetic precursors and energy to support viral replication (20, 21). Together, these studies highlight mitophagy modulation, whether fully executed or deliberately stalled, as a conserved viral strategy to rewire host metabolism and create a replication-friendly intracellular niche. In crustaceans, however, it remains unknown whether a Drp1- and PINK1–Parkin-mediated mitochondrial quality control pathway exists and participates in host–virus interactions. Even if such a pathway is present, it is unclear whether WSSV fully hijacks it or selectively modulates specific steps to benefit viral replication. Addressing these questions is essential for a comprehensive understanding of WSSV pathogenesis.

In this study, we reveal that WSSV infection hijacks host mitochondrial quality control via the viral protein wsv156, which triggers *Cq*Drp1-dependent mitochondrial aggregation, thereby activating the conserved PINK1–Parkin mitophagy pathway. Notably, the virus does not drive conventional clearance-type autophagy; instead, it establishes an incomplete autophagic state that enhances glycolysis and suppresses apoptosis to promote viral replication. Our findings identify wsv156 as a key molecular switch and reveal an evolutionarily conserved strategy whereby large DNA viruses manipulate organellar quality control across diverse hosts, offering new insights into viral pathogenesis and potential therapeutic targets.

## RESULTS

### WSSV infection induces mito-aggresome formation

**Fig 1:**
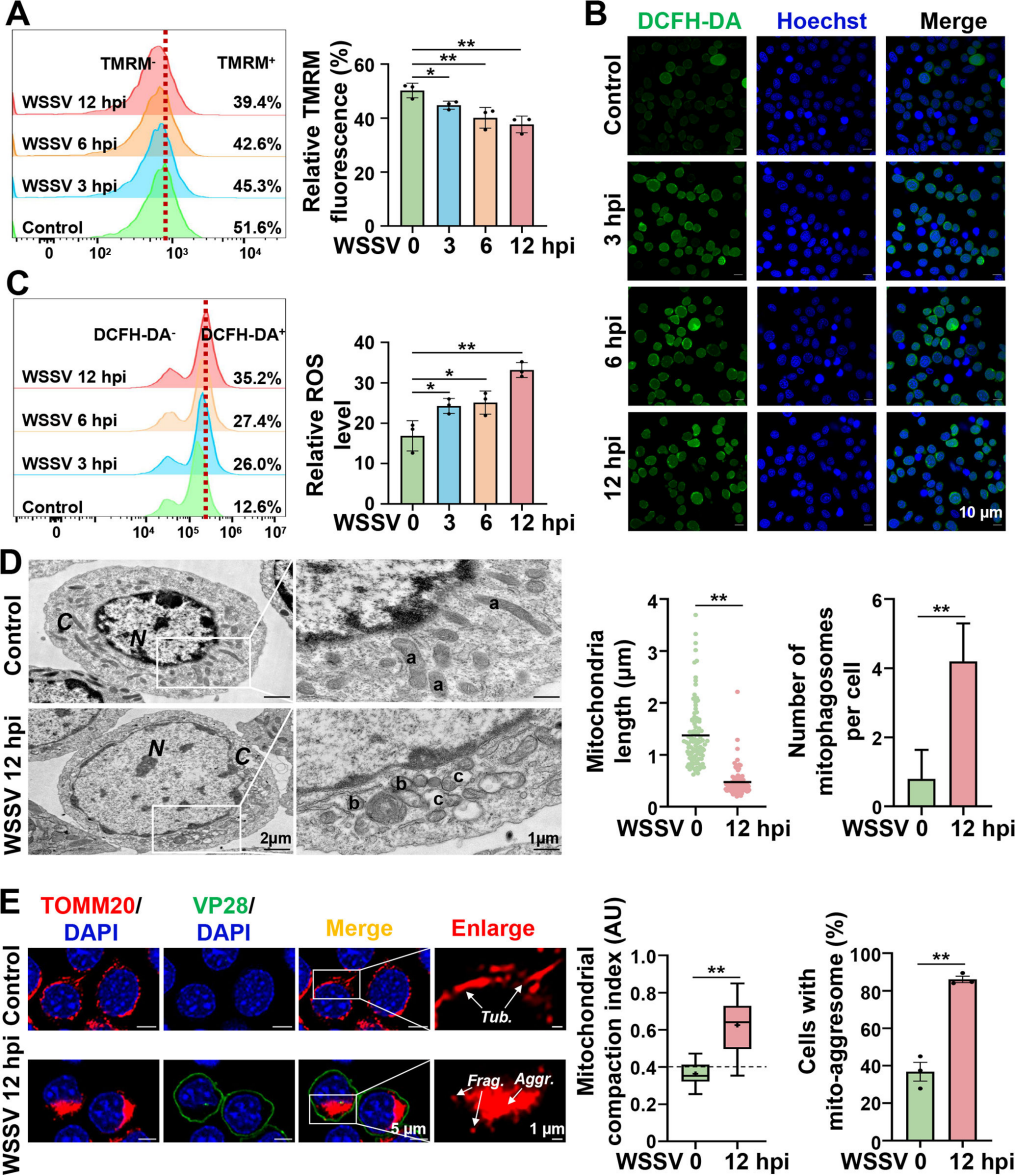
WSSV infection induces mitochondrial damage and mito-aggresome formation. (**A**) WSSV infection induced mitochondrial membrane depolarization. The mitochondrial membrane potential was assessed by measuring relative TMRM intensity via flow cytometry in Hpt cells (left panel). Statistical analysis of the results is shown in the right panel. (B and C) WSSV infection promoted ROS generation. ROS levels in Hpt cells were evaluated using DCFH-DA staining, as examined by confocal fluorescence microscopy (**B**) or flow cytometry (**C**). Statistical analysis of ROS fluorescence (carboxy-DCF) measured by flow cytometry is shown in the right panel. (**D**) WSSV infection induced mitochondrial damage. Compared to mitochondria in negative control Hpt cells, which displayed clear cristae and intact double membranes (a), mitochondria from WSSV-infected cells showed structural damage, including loss of inner membranes and cristae (b), and autophagic vacuoles containing mitochondria (c). Quantitative analyses of mitochondrial length and the number of mitophagosomes per cell are presented in the middle and right panels, respectively. *C*, cytoplasm; *N*, nucleus. (**E**) WSSV infection triggered mitochondrial aggregation. Whereas mitochondria in control Hpt cells exhibited an elongated, tubular morphology (upper panel), prominent mito-aggresomes were observed in WSSV-infected Hpt cells (lower panel), as visualized by immunostaining against the mitochondrial outer membrane protein TOMM20 (red). WSSV-infected Hpt cells were labeled with an antibody against the viral envelope protein VP28 (green). Nuclei were stained with DAPI (blue). Tub., tubular mitochondria; Frag., fragmented mitochondria; Aggr., aggregated mitochondria. The middle panel shows the mitochondrial aggregation assessed by the CI, and the right panel presents the percentage of Hpt cells containing mito-aggresomes. All statistical analyses were performed based on at least three independent experiments. Significant differences were analyzed using Student’s *t* test: **, *P* < 0.01; *, *P* < 0.05.

To determine whether WSSV infection induces mitochondrial dysfunction, we measured mitochondrial membrane potential (MMP, expressed as ΔΨm) in crayfish hematopoietic tissue (Hpt) cells using the potentiometric dye tetramethylrhodamine methyl ester (TMRM). Flow cytometry revealed a progressive and significant decrease in MMP at 3, 6, and 12 h post-infection (hpi) compared to mock-infected cells (Fig. 1A). Concurrently, reactive oxygen species (ROS) production increased, as shown by enhanced green fluorescence in infected Hpt cells via fluorescence microscopy and flow cytometry (Fig. 1B and C). These data together indicate that WSSV infection induces mitochondrial dysfunction. Transmission electron microscopy (TEM) further revealed that at 12 hpi, mitochondrial length was significantly shortened and the number of mitophagosomes increased (Fig. 1D), suggesting ultrastructural damage to mitochondria. Notably, damaged mitochondria appeared as tightly clustered aggregates, a morphology consistent with previous reports that viral infection can induce mito-aggresomes (17). Consistent with these TEM observations, immunofluorescence staining of the mitochondrial outer-membrane protein *Cq*TOMM20 showed prominent perinuclear clustering of mitochondria in infected Hpt cells at 12 hpi, contrasting with the diffuse mitochondrial distribution in uninfected controls; quantification indicated a significant rise in the mitochondrial compaction index (CI), with >80% of infected cells exhibiting mito-aggresomes (Fig. 1E). Together, these findings demonstrate that WSSV infection triggers mitochondrial dysfunction that is closely accompanied by the formation of mito-aggresomes.

### Wsv156 targets mitochondria via *Cq*TOMM70 and induces mito-aggresome formation

**Fig 2:**
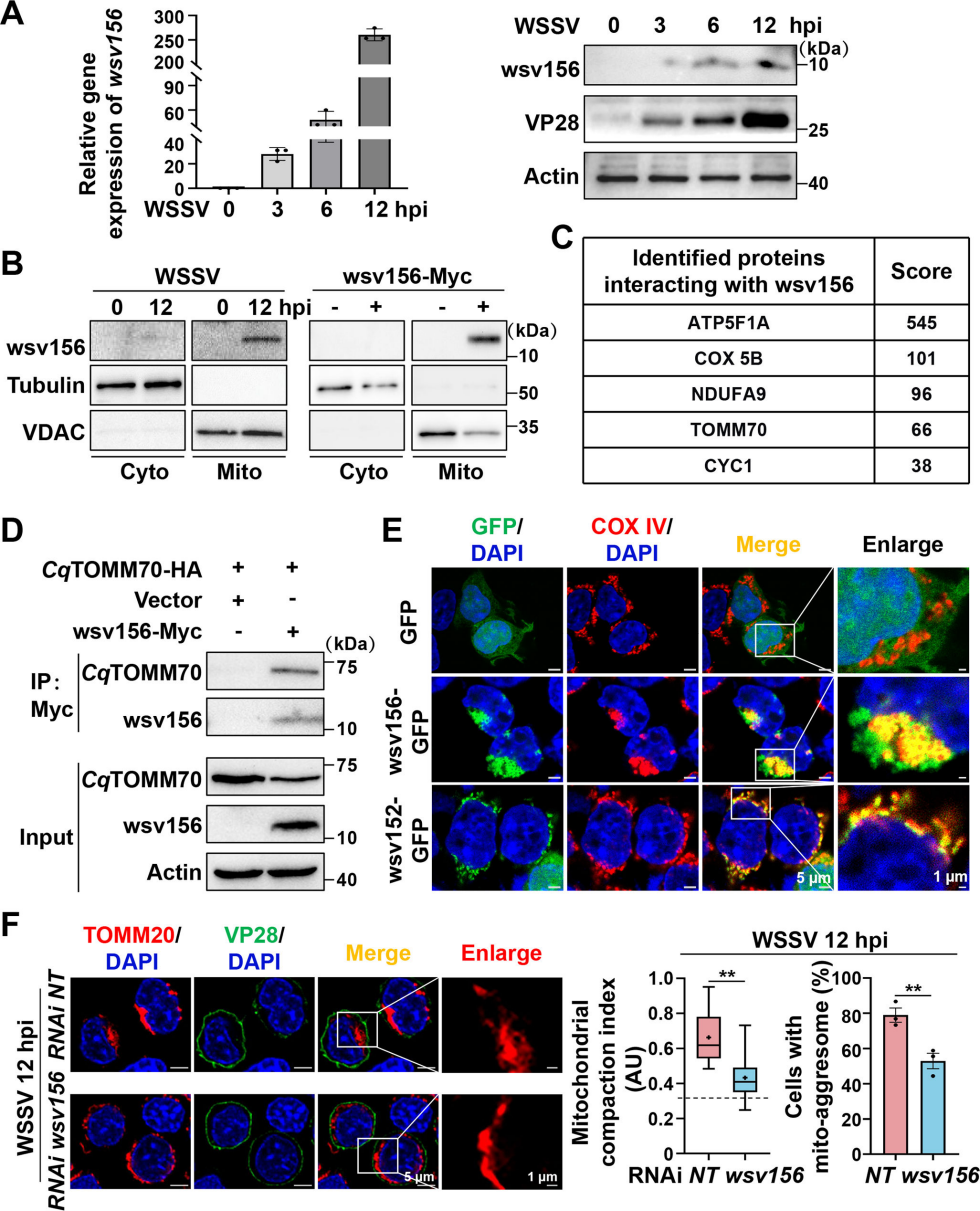
Wsv156 targets mitochondria via *Cq*TOMM70 and triggers mito-aggresome formation. (**A**) Wsv156 expression increased following WSSV infection. Temporal expression of wsv156 at the mRNA (left panel) and protein level (right panel) was assessed by RT-qPCR and western blot, respectively, in Hpt cells after WSSV infection. (**B**) Wsv156 localized to mitochondria. Endogenous wsv156 protein in Hpt cells (left panel) and overexpressed wsv156 in HEK 293T cells (right panel) both predominantly co-fractionated with mitochondria. (**C**) Identification of host mitochondrial proteins interacting with wsv156 by affinity purification-mass spectrometry (AP-MS). (**D**) Wsv156 interacted with *Cq*TOMM70. Co-IP with anti-Myc beads was performed in HEK 293T cells co-transfected with wsv156-Myc and *Cq*TOMM70-HA. (**E**) Wsv156 expression induced mitochondrial aggregation. Confocal microscopy of HEK 293T cells showed co-localization of wsv156-GFP (green) with the mitochondrial marker *Hs*COX IV (red). Cells expressing GFP alone or wsv152-GFP served as controls. (**F**) *Wsv156* knockdown attenuated WSSV-induced mitochondrial aggregation. Gene silencing of *wsv156* in Hpt cells reduced mitochondrial clustering (red) compared to control cells at 12 hpi. NT, non-targeting dsRNA (GFP dsRNA) control; RNAi wsv156, wsv156-specific dsRNA. The right panel quantifies mitochondrial aggregation using the CI and the percentage of cells containing mito-aggresomes. Nuclei were stained with DAPI. **, *P* < 0.01.

After observing that WSSV infection promotes mitochondrial dysfunction, prominently manifested as mito-aggresome formation, we aimed to identify the viral factors responsible for these mitochondrial changes. We first screened all 532 WSSV ORFs using MitoProt software, which identified 45 candidates with high predicted mitochondrial localization. These ORFs were cloned and expressed in HEK 293T cells; confocal microscopy analysis revealed 12 viral proteins that exhibited clear mitochondrial targeting (data not shown). Among these, *wsv156* was selected for further study because its expression alone phenocopied the mitochondrial aggregation phenotype observed during WSSV infection. MitoProt II analysis predicted mitochondrial localization with a high probability score of 0.9. Moreover, the predicted N-terminal amphipathic α-helix (residues 1–25) showed a clear segregation of basic and hydrophobic residues into opposing faces, a hallmark of classical mitochondrial presequences (Fig. S1) (22). Supporting a potential early role in infection, *wsv156* transcripts were detectable at 3 hpi and increased substantially throughout the viral replication cycle, remaining elevated until at least 12 hpi (Fig. 2A), consistent with an early viral gene expression profile. Subcellular fractionation of WSSV-infected Hpt cells at 12 hpi, followed by western blot with an anti-wsv156 polyclonal antibody, showed wsv156 predominantly in the mitochondrial fraction, with little signal in the cytosol (Fig. 2B, left panel). This mitochondrial localization was further confirmed in HEK 293T cells overexpressing wsv156-Myc, where anti-Myc immunoblotting detected wsv156 exclusively in mitochondrial isolates (Fig. 2B, right panel). Together, these data establish wsv156 as a mitochondria-localized viral protein during infection.

To elucidate the mitochondrial targeting mechanism of wsv156, we employed mass spectrometry analysis, which identified mitochondrial proteins including ATP synthase F1 subunit alpha (ATP5F1A), cytochrome c oxidase subunit 5B (COX5B), NADH: ubiquinone oxidoreductase subunit A9 (NDUFA9), outer mitochondrial membrane protein 70 (TOMM70), and cytochrome c1 (CYC1) (Fig. 2C). Given the established role of the outer mitochondrial membrane translocase TOMM70 in mediating protein import into mitochondria (23), we selected TOMM70 for further interaction studies. Co-immunoprecipitation (Co-IP) assays confirmed a direct and specific interaction between Myc-tagged wsv156 and HA-tagged *Cq*TOMM70 (Fig. 2D), establishing *Cq*TOMM70 as a key interacting partner for the mitochondrial import of this viral protein. To determine the functional consequence of the wsv156-TOMM70 interaction, we expressed GFP-tagged wsv156 in HEK 293T cells, which induced pronounced mitochondrial aggregation, as shown by staining with the mitochondrial marker cytochrome c oxidase subunit IV (COX IV). In contrast, cells expressing the mitochondrial-targeted control protein GFP-tagged wsv152 maintained normal mitochondrial morphology (Fig. 2E). Correspondingly, in Hpt cells, knockdown of *wsv156* strongly suppressed the mitochondrial aggregation triggered by WSSV infection (Fig. 2F), demonstrating that wsv156 is essential for virus-induced mito-aggresomes formation.

### Wsv156 drives *Cq*Drp1-dependent mito-aggresomes formation

**Fig 3:**
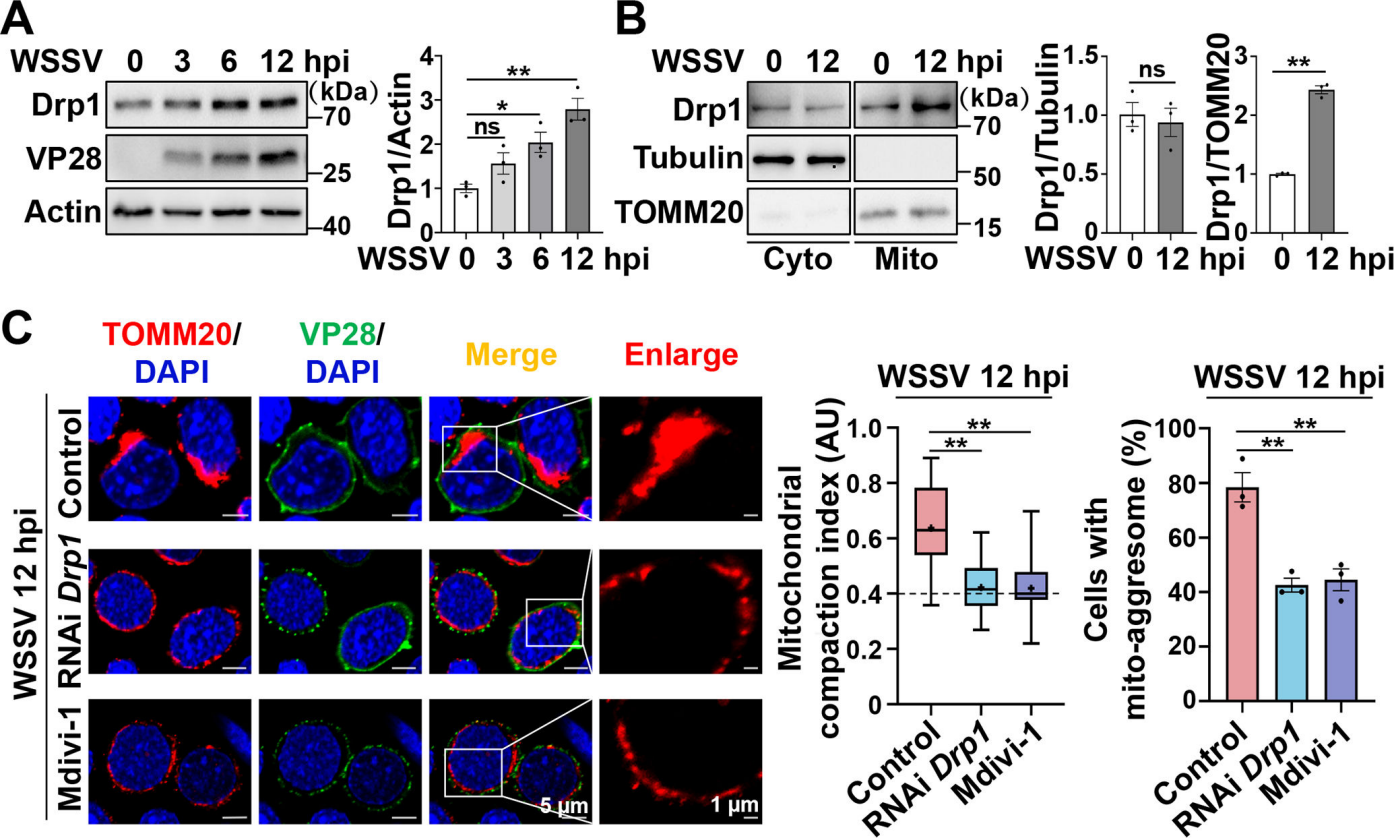
*Cq*Drp1 is required for WSSV-induced mito-aggresome formation. (**A**) WSSV infection upregulated endogenous *Cq*Drp1 protein levels. The increase in *Cq*Drp1 protein upon WSSV infection was detected by western blot (left panel) and statistically quantified (right panel). (**B**) WSSV infection promoted mitochondrial translocation of *Cq*Drp1. The protein ratio of *Cq*Drp1 to the cytosolic marker *Cq*Tubulin (cytosolic fraction) or to the mitochondrial marker *Cq*TOMM20 (mitochondrial fraction) was analyzed. *Cq*Drp1 showed significant enrichment in the mitochondrial fraction following WSSV infection, as statistically demonstrated in the right panel. (**C**) Inhibition of *Cq*Drp1 attenuated WSSV-induced mito-aggresome formation. *Cq*Drp1 was inhibited either by RNAi-mediated gene silencing or pharmacologically using Mdivi-1 in WSSV-infected Hpt cells. Cells were immunostained with antibodies against TOMM20 (mitochondria, red) and VP28 (WSSV, green); and nuclei were counterstained with DAPI (blue). The right panel presents the quantitative analysis of the mitochondrial CI and the percentage of Hpt cells containing mito-aggresomes. All statistical analyses were performed based on at least three independent experiments. Significant differences were analyzed using Student’s *t* test: **, *P* < 0.01; *, *P* < 0.05; ns, no significant difference.

We next investigated how mitochondria-localized wsv156 induces mito-aggresome formation. To explore the molecular basis of WSSV-induced mito-aggresome formation in crayfish (Fig. 1), we analyzed the expression and localization of *Cq*Drp1 (GenBank: XM_070094146.1), an evolutionarily conserved GTPase of mitochondrial fission and aggregation in mammals and insects (24). Western blot analysis showed that *Cq*Drp1 progressively increased in Hpt cells at 3, 6, and 12 hpi during WSSV infection (Fig. 3A). Moreover, *Cq*Drp1 levels were markedly elevated in the mitochondrial fraction of infected cells, confirming its mitochondrial translocation and supporting a role in infection-triggered mitochondrial aggregation (Fig. 3B). Given the observed increase in mito-aggresome formation after WSSV infection (Fig. 1E), we next asked whether Drp1 activity is functionally required. Interestingly, knockdown of *CqDrp1* or treatment with the Drp1-specific inhibitor Mdivi-1, a compound that targets mitochondrial division, significantly suppressed mito-aggresome formation at 12 hpi (Fig. 3C, left panel); quantification revealed a significant reduction in both the CI values and the percentage of Hpt cells containing mito-aggresomes (Fig. 3C, right panel), indicating that *Cq*Drp1 is essential for WSSV-induced mito-aggresome formation.

**Fig 4:**
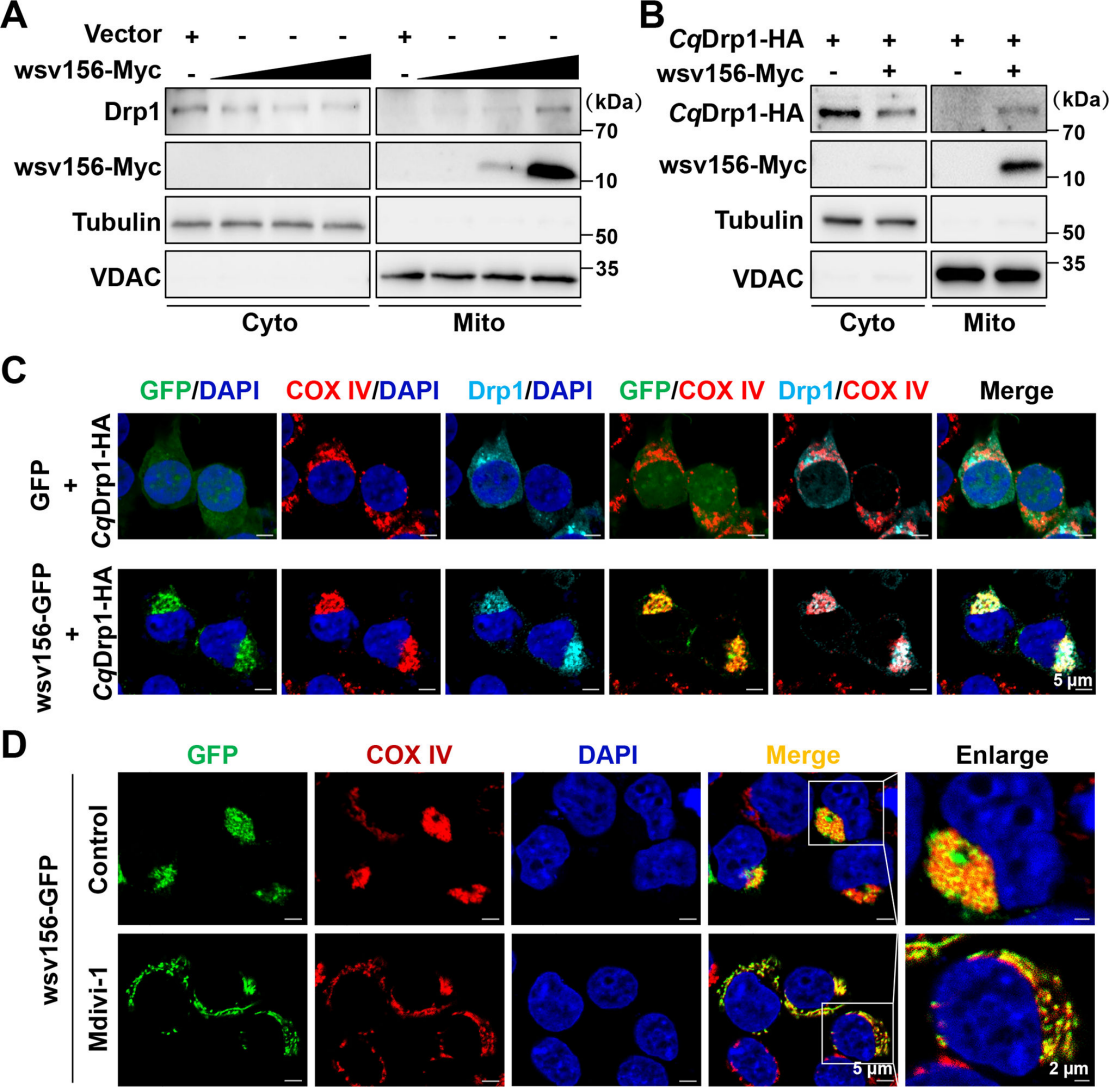
Wsv156-induced *Cq*Drp1-dependent mito-aggresome formation. (**A and B**) Wsv156 induced Drp1 translocation to mitochondria. Wsv156-Myc expression in HEK 293T cells increased mitochondrial localization of both endogenous *Hs*Drp1 (**A**) and overexpressed *Cq*Drp1 (**B**), as shown by western blot analysis of mitochondrial fractions. (**C**) Wsv156 enhanced *Cq*Drp1 mitochondrial accumulation. Immunofluorescence against COX IV (mitochondria, red) in HEK 293T cells confirmed increased mitochondrial co-localization of *Cq*Drp1 upon wsv156 expression. (**D**) Pharmacological inhibition of Drp1 suppressed wsv156-induced mito-aggresome formation. While wsv156-GFP expression triggered pronounced mitochondrial aggregates (labeled with anti-COX IV, red), treatment with the Drp1 inhibitor Mdivi-1 significantly reduced aggregate formation in HEK 293T cells.

Given that wsv156 was found to induce mito-aggresome formation, we next sought to determine whether *Cq*Drp1 is required for this process directly triggered by wsv156. The cytosolic and mitochondrial fractions were then isolated from wsv156-Myc-overexpressing HEK 293T cells. Notably, the endogenous Drp1 protein levels were elevated in the mitochondrial fraction but not in the cytosolic fraction of HEK 293T cells (Fig. 4A). This mitochondrial recruitment of Drp1 was further confirmed in cells co-overexpressing wsv156 and *Cq*Drp1-HA, where wsv156 enhanced *Cq*Drp1-HA accumulation in mitochondria (Fig. 4B). Immunofluorescence analysis also showed enhanced co-localization of *Cq*Drp1 with the mitochondrial marker COX IV in wsv156-expressing cells (Fig. 4C). These data indicated that wsv156 promoted Drp1 translocation to mitochondria. Next, to determine whether *Cq*Drp1 translocation was required for mitochondrial aggregation, we performed immunofluorescence analysis. The results showed that pretreatment with Drp1 inhibitor Mdivi-1 effectively suppressed the formation of wsv156-induced mito-aggresomes (Fig. 4D). In control cells (expressing wsv156 and treated with DMSO), prominent mitochondrial aggregation and peri-nuclear clustering were evident (Fig. 4D, top); in contrast, in cells pretreated with Mdivi-1, mitochondria displayed a more diffuse cytoplasmic distribution, with significantly reduced aggregation (Fig. 4D, bottom). Taken together, these data establish that *Cq*Drp1 is required for wsv156-dependent mito-aggresome formation.

### WSSV infection triggers *Cq*Parkin-dependent mitophagy via its protein wsv156

**Fig 5:**
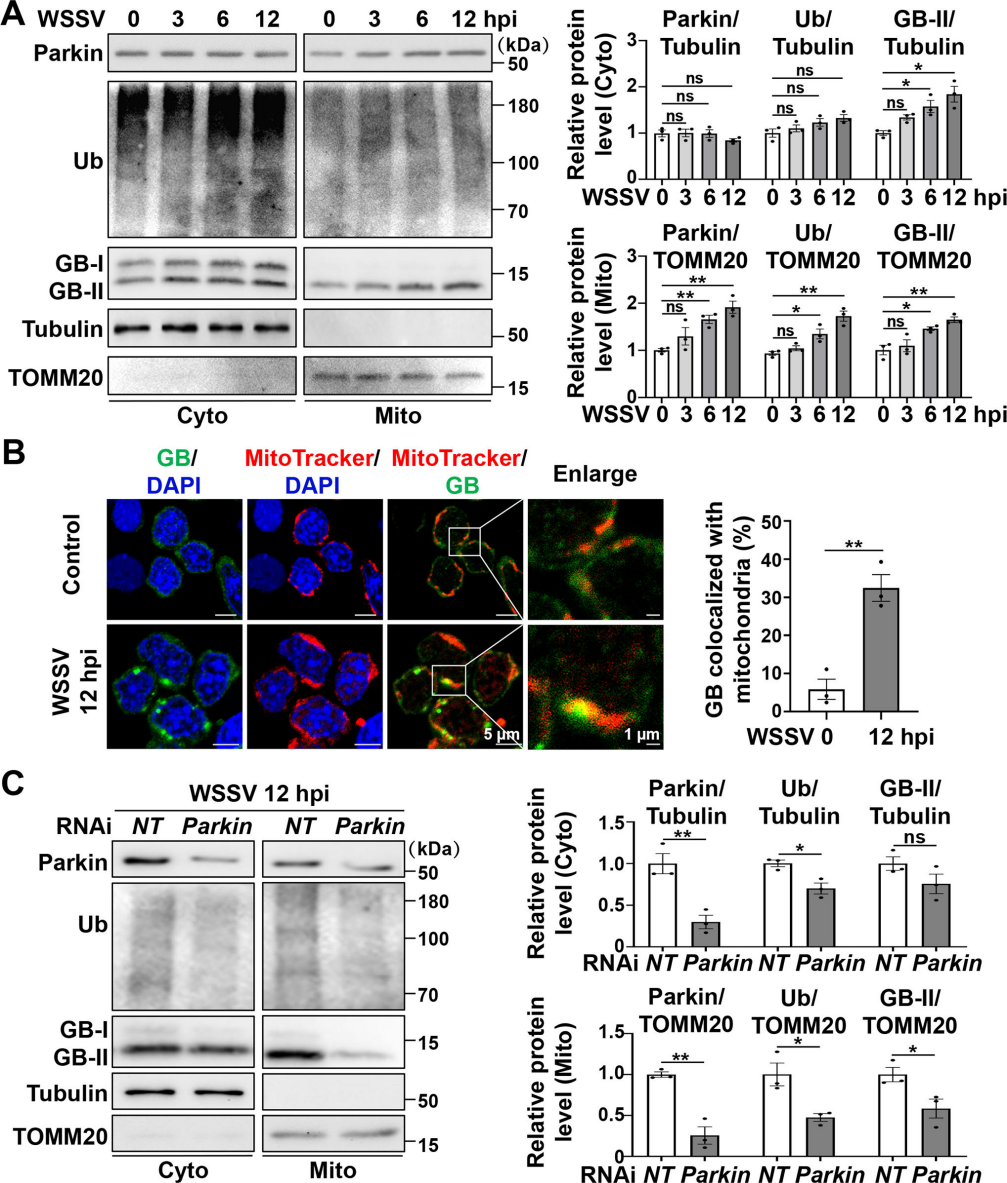
WSSV infection triggers *Cq*Parkin-dependent mitophagy. (**A**) WSSV infection enhanced mitophagy initiation. Levels of *Cq*Parkin, ubiquitinated proteins, and lipidated *Cq*GB-II (markers of mitophagy initiation) were elevated in the mitochondrial fraction from Hpt cells after WSSV infection. Statistical analysis of these changes is shown in the right panel. (**B**) WSSV infection promoted mitophagic sequestration of mitochondria. Hpt cells were stained with MitoTracker Red (mitochondria), anti-GB antibody (green), nuclei were counterstained with DAPI (blue). The right panel shows quantitative analysis of GB-positive puncta co-localizing with mitochondria. (**C**) *Cq*Parkin was required for mitochondrial recruitment of ubiquitinated proteins and *Cq*GB-II. Mitochondrial and cytosolic fractions from Hpt cells after WSSV infection were isolated using *Cq*TOMM20 and *Cq*Tubulin as respective markers. The protein ratios were statistically analyzed, as shown in the right panel. All statistical analyses were performed based on at least three independent experiments. Significant differences were analyzed using Student’s *t* test: **, *P* < 0.01; *, *P* < 0.05; ns, no significant difference.

Damaged mitochondria are selectively removed via mitophagy, a process primarily governed by the PINK1–Parkin pathway. Parkin, an E3 ubiquitin ligase, is recruited to depolarized mitochondria where it ubiquitinates outer-membrane proteins, thereby tagging mitochondria for autophagic clearance (13). To examine whether WSSV-induced mito-aggresomes activate mitophagy, we identified and characterized *CqPINK1* (GenBank: XM_070097991.11) and *CqParkin* (GenBank: XM_053772764.2) in the red claw crayfish. Western blot analysis of mitochondrial fractions from WSSV-infected Hpt cells revealed pronounced accumulation of *Cq*Parkin, ubiquitinated proteins, and the ATG8/LC3 family protein γ-aminobutyric acid receptor-associated protein (*Cq*GABARAP, *Cq*GB)-II (Fig. 5A). Consistent with this, confocal microscopy showed enhanced co-localization of mitochondria with *Cq*GB-II puncta in WSSV-infected Hpt cells (Fig. 5B), together indicating that WSSV infection activated mitophagy. To determine whether this process depends on *Cq*Parkin, we silenced *CqParkin* in Hpt cells. Mitochondrial fractions from *CqParkin*-silenced cells collected at 12 hpi exhibited markedly lower levels of ubiquitinated proteins and *Cq*GB-II compared with control-infected cells (Fig. 5C). This result corroborates the conserved role of Parkin as an E3 ubiquitin ligase that marks damaged mitochondria for autophagic degradation, confirming that WSSV-induced mitophagy in crustacean cells proceeds via a *Cq*Parkin-dependent pathway.

**Fig 6:**
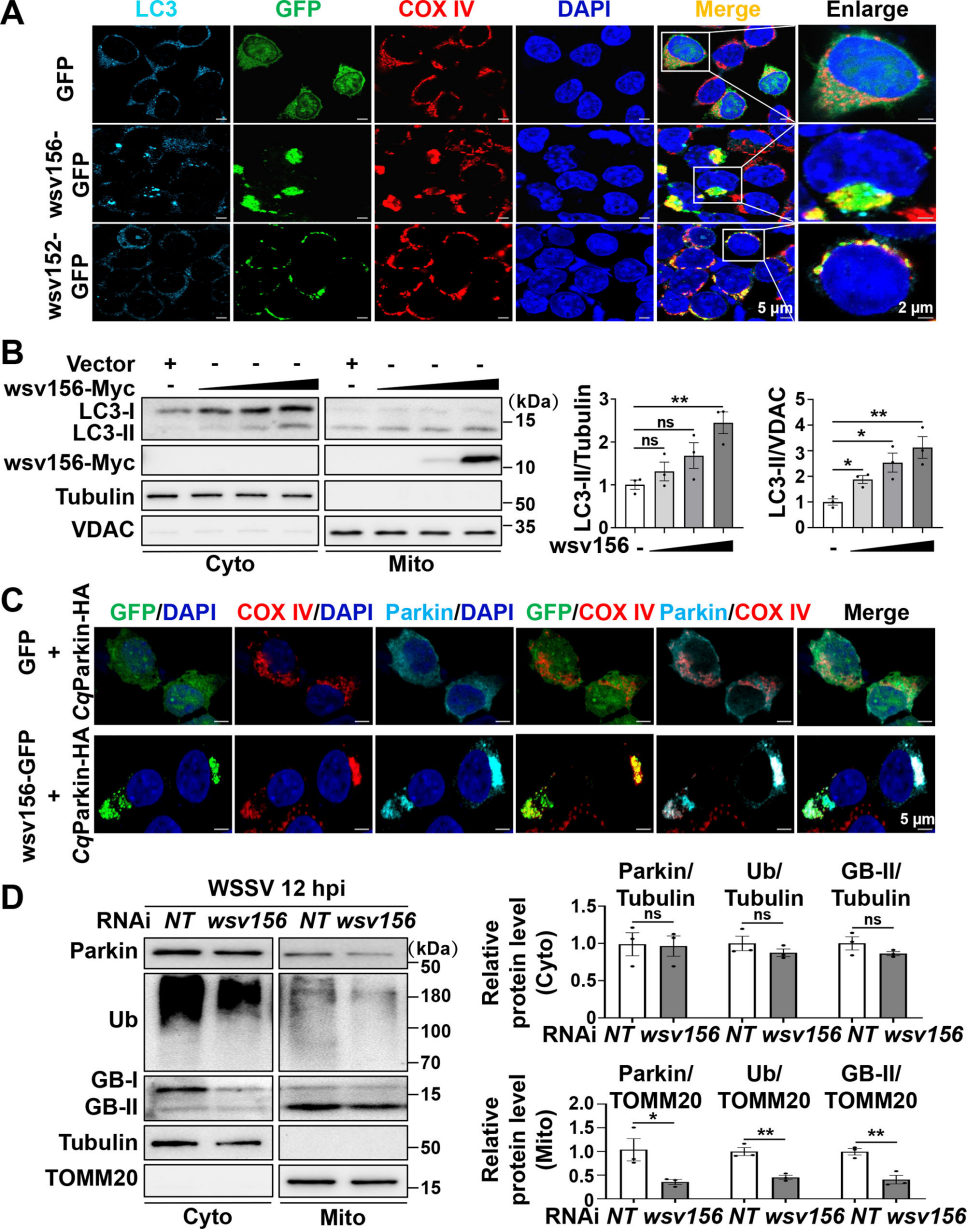
Wsv156 induces *Cq*Parkin-dependent mitophagy. (**A**) Wsv156 mitochondrial localization induced LC3 puncta accumulation. Compared to control HEK 293T cells expressing GFP or wsv152-GFP, which displayed few LC3 puncta (turquoise), wsv156-expressing cells showed significantly increased LC3 puncta co-localizing with mitochondria labeled by *Hs*COX IV (red). (**B**) Wsv156 promoted mitochondrial enrichment of lipidated LC3-II. Mitochondrial and cytosolic fractions from HEK 293T cells were analyzed using *Hs*VDAC and *Hs*Tubulin as respective markers. The protein ratio of *Hs*LC3-II to *Hs*Tubulin or *Hs*VDAC was quantified and shown in the right panel. (**C**) Wsv156 enhanced Parkin localization to mitochondria. In HEK 293T cells overexpressing wsv156-GFP (green), *Cq*Parkin-HA (turquoise) strongly co-localized with the mitochondrial marker *Hs*COX IV (red), whereas control cells without wsv156 expression showed no such co-localization. (**D**) *Wsv156* knockdown impaired *Cq*Parkin-mediated mitophagy. Gene silencing of *wsv156* in Hpt cells suppressed mitochondrial translocation of *Cq*Parkin, and consequently reduced accumulation of ubiquitinated proteins and *Cq*GB-II, as shown by western blot (left panel) and quantified statistically (right panel). All statistical analyses were performed based on at least three independent experiments. Significant differences were analyzed using Student’s *t* test: **, *P* < 0.01; *, *P* < 0.05; ns, no significant difference.

To investigate how WSSV engages this pathway, we explored whether wsv156, the viral inducer of mitochondrial aggregation, regulates *Cq*Parkin-dependent mitophagy. We first assessed the recruitment of LC3, a core autophagy marker, to mitochondria. Immunofluorescence analysis revealed that wsv156 overexpression led to a pronounced accumulation of LC3 puncta that extensively colocalized with mitochondria in HEK 293T cells, whereas wsv152 overexpression did not elicit a comparable phenotype (Fig. 6A). Immunoblotting further confirmed elevated LC3-II levels in mitochondrial fractions from HEK 293T cells (Fig. 6B), indicating specific recruitment of autophagic components to mitochondria and initiation of mitophagosome formation. Although these observations confirmed wsv156-induced mitophagy activation, the upstream mechanism mediating mitochondrial recognition remains unclear. Given that WSSV-induced mitophagy requires *Cq*Parkin (Fig. 5), we next investigated whether wsv156 directly recruits *Cq*Parkin to mitochondria to trigger mitophagy initiation. HEK 293T cells were co-transfected with *Cq*Parkin and wsv156-GFP and subjected to immunofluorescence analysis. In control cells expressing *Cq*Parkin and GFP, *Cq*Parkin showed a diffuse cytoplasmic distribution, with no evident mitochondrial aggregation or specific *Cq*Parkin recruitment (Fig. 6C, upper); while in cells co-transfected with both *Cq*Parkin and wsv156-GFP, wsv156 was predominantly localized to mitochondria and strongly promoted mitochondrial aggregation, and a pronounced co-localization signal between *Cq*Parkin and mitochondria was detected, indicating that wsv156 enhances the recruitment of *Cq*Parkin to mitochondria (Fig. 6C, lower). Notably, in WSSV-infected Hpt cells, gene silencing of *wsv156* inhibited mitochondrial recruitment of *Cq*Parkin and reduced the accumulation of both ubiquitinated proteins and *Cq*GB-II in the mitochondrial fraction, compared with control cells (Fig. 6D). Together, these results demonstrate that *Cq*Parkin plays an essential role in mediating wsv156-induced mitophagy.

### The mitochondria-targeted wsv156 triggers complete mitophagy

**Fig 7:**
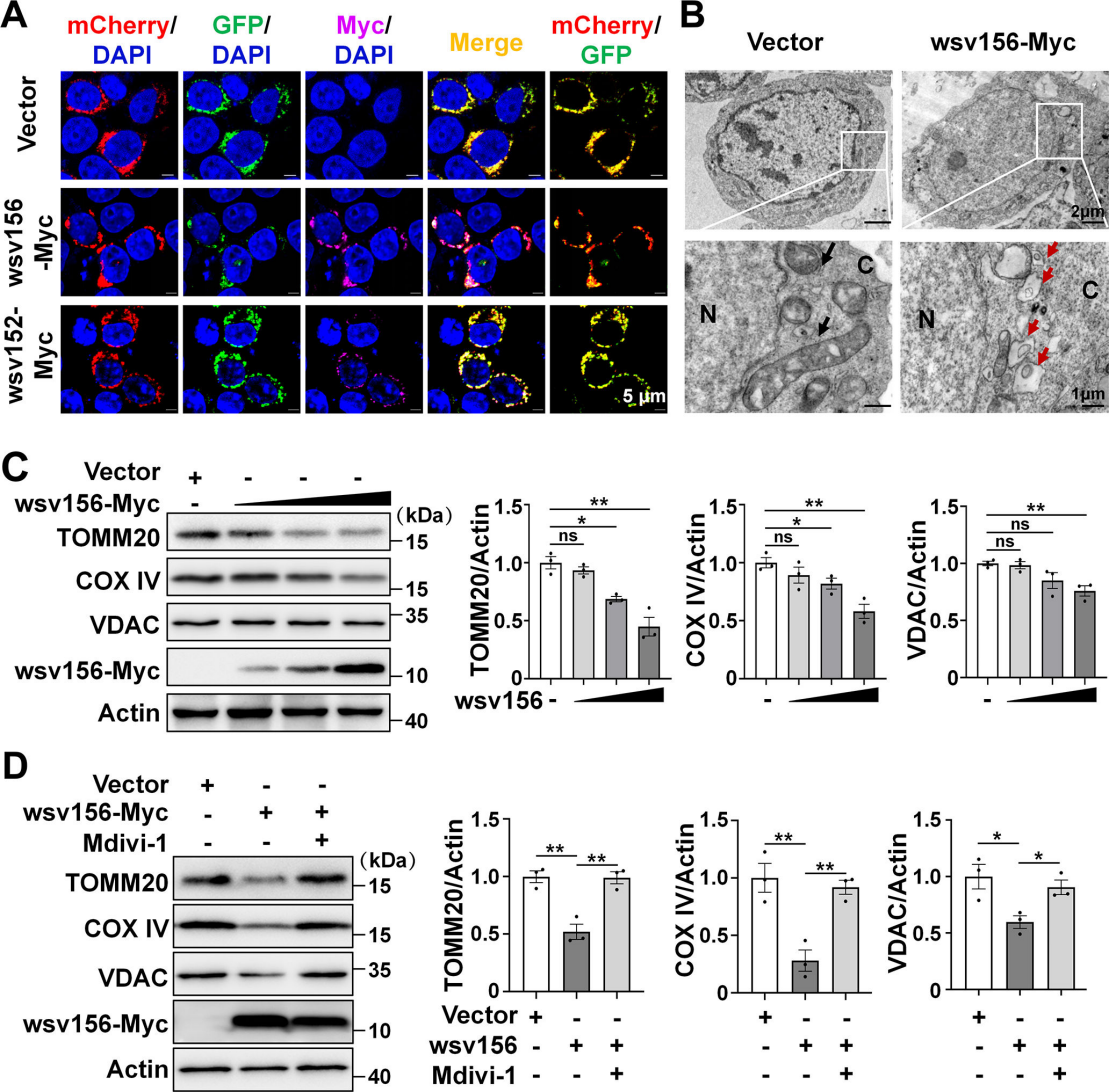
Mitochondrial targeting of wsv156 triggers complete mitophagy. (**A**) Wsv156 enhanced mitophagosome–lysosome fusion. Using the Mito-mCherry-GFP reporter, wsv156-expressing cells exhibited a marked increase in red-only puncta, indicating acidified mitolysosomes (GFP quenched in acidic compartments). No such increase was observed in control cells expressing wsv152. (**B**) Wsv156 increased mitophagic vacuole formation. Transmission electron microscopy of HEK 293T cells expressing wsv156-Myc revealed an increased number of mitophagic vacuoles (red arrows) compared with controls. Mitochondria are indicated by black arrows. *C*, cytoplasm; *N*, nucleus. (**C**) Wsv156 promoted complete mitophagy. Mitochondrial protein levels of *Hs*TOMM20, *Hs*COX IV, and *Hs*VDAC were reduced in HEK 293T cells overexpressing wsv156. Densitometric analysis of three biological replicates is shown in the right panel. (**D**) Drp1 inhibition attenuated wsv156-induced mitophagy. Degradation of mitochondrial proteins (*Hs*TOMM20, *Hs*COX IV, and *Hs*VDAC) induced by wsv156 was blocked when HEK 293T cells were treated with Mdivi-1. All statistical analyses were performed based on at least three independent experiments. Significant differences were analyzed using Student’s *t* test: **, *P* < 0.01; *, *P* < 0.05; ns, no significant difference.

The recruitment and activation of Parkin typically initiate the labeling of damaged mitochondria for autophagic clearance. To determine whether wsv156-activated mitophagy proceeds to completion, specifically, whether labeled mitochondria are successfully delivered to and degraded in lysosomes, mitophagic flux was measured directly. We employed a dual-fluorescence Mito-mCherry-GFP reporter, in which GFP fluorescence is quenched upon lysosomal acidification, resulting in red-only puncta that indicate completed mitophagy. HEK 293T cells expressing wsv156 exhibited abundant red-only puncta, indicating efficient lysosomal delivery and consequently complete mitophagic flux (Fig. 7A). In contrast, cells expressing the mitochondria-targeted control protein wsv152, which lacks mitophagy-inducing activity, resulted in predominantly yellow fluorescence, indicative of intact mitochondria with no induction of mitophagy (Fig. 7A). Furthermore, TEM of HEK 293T cells transfected with wsv156-Myc plasmid confirmed the sequestration of mitochondria within autophagosomes (Fig. 7B). Correspondingly, western blot analysis showed that mitochondrial targeting of wsv156 significantly reduced the levels of key mitochondrial proteins (*Hs*TOMM20, *Hs*COX IV, and *Hs*VDAC), confirming mitochondrial degradation (Fig. 7C). To determine whether Drp1-mediated mitochondrial aggregation is required for wsv156-induced mitophagy, we analyzed the levels of mitochondrial proteins *Hs*TOMM20 and *Hs*COX IV by western blot. The results showed that Drp1 inhibition by Mdivi-1 pretreatment blocked the degradation of these proteins in wsv156-expressing cells (Fig. 7D), demonstrating that Drp1 activity is essential for the progression of wsv156-induced mitophagy. Taken together, our data establish that the mitochondrial targeting of wsv156 drives complete mitophagy. The precise relationship between observed mitochondrial aggregation and the initiation of mitophagy, however, warrants further investigation.

### Wsv156-triggered mitophagy is diverted toward an incomplete state by viral VP26

**Fig 8:**
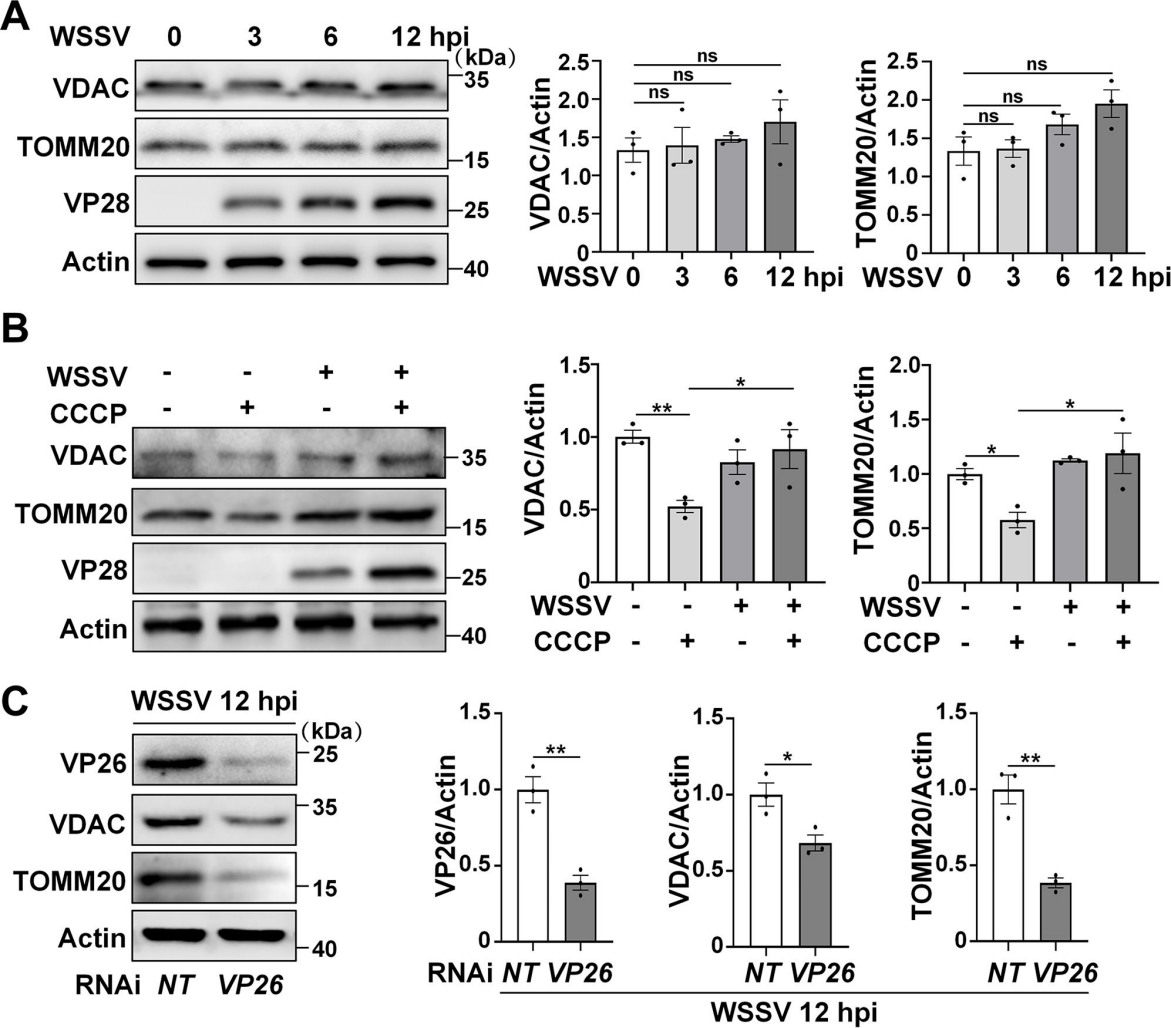
WSSV-triggered mitophagy is diverted toward an incomplete state by viral VP26. (**A**) WSSV infection inhibited mitophagic degradation. Mitochondrial proteins *Cq*VDAC and *Cq*TOMM20 were not degraded in Hpt cells after WSSV infection. The relative quantitative ratio of *Cq*VDAC or *Cq*TOMM20 to *Cq*Actin is shown in the right panel. (**B**) WSSV infection blocked mitophagic flux. The CCCP-induced mitophagic flux, reflected by degradation of *Cq*VDAC and *Cq*TOMM20, was blocked by WSSV infection. The relative quantitative ratio of *Cq*VDAC or *Cq*TOMM20 to *Cq*Actin is shown in the right panel. (**C**) *VP26* silencing restored mitophagic degradation. Gene silencing of *VP26* promoted the degradation of mitochondrial proteins *Cq*VDAC and *Cq*TOMM20 in crayfish hematopoietic tissue *in vivo*, indicating that VP26 inhibits mitophagy completion. The relative quantitative ratio of *Cq*VDAC or *Cq*TOMM20 to Actin is shown in the right panel. All statistical analyses were performed based on at least three independent experiments. Significant differences were analyzed using Student’s *t* test: **, *P* < 0.01; *, *P* < 0.05; ns, no significant difference.

Our findings demonstrate that the mitochondria-targeted viral protein wsv156 alone is sufficient to induce complete mitophagy (Fig. 7). However, it remained unclear whether this process is fully executed during WSSV infection, or whether the virus employs additional regulatory mechanisms to modulate its progression. To evaluate the status of mitophagy in infected cells, we assessed the levels of mitochondrial marker proteins *Cq*TOMM20 and *Cq*VDAC in Hpt cells. Western blot analysis showed no significant degradation of these proteins at 3, 6, or 12 hpi (Fig. 8A). Moreover, the mitophagy activator carbonyl cyanide m-chlorophenyl hydrazone (CCCP)-induced degradation of these mitochondrial proteins was significantly impaired in WSSV-infected cells compared to uninfected controls (Fig. 8B), indicating that the virus induces incomplete mitophagy. Given our previous finding that the WSSV envelope protein VP26 inhibits autophagosome-lysosome fusion and blocks autophagic flux, thereby promoting viral replication (11), we investigated whether VP26 similarly interferes with mitophagy completion. Western blot analysis of Hpt tissue showed a strong reduction in VP26 protein via gene silencing of *VP26* in WSSV-infected crayfish *in vivo*, which was accompanied by a significant decrease in *Cq*VDAC and *Cq*TOMM20 levels (Fig. 8C), indicating that *VP26* knockdown restored mitochondrial degradation. These results demonstrate that WSSV activates the *Cq*Parkin-dependent mitophagy pathway through wsv156 but concurrently blocks its completion via the envelope protein VP26. This coordinated “initiate-but-not-complete” strategy leads to a sustained state of incomplete mitophagy during infection.

### Incomplete mitophagy induced by WSSV promotes viral infection

**Fig 9:**
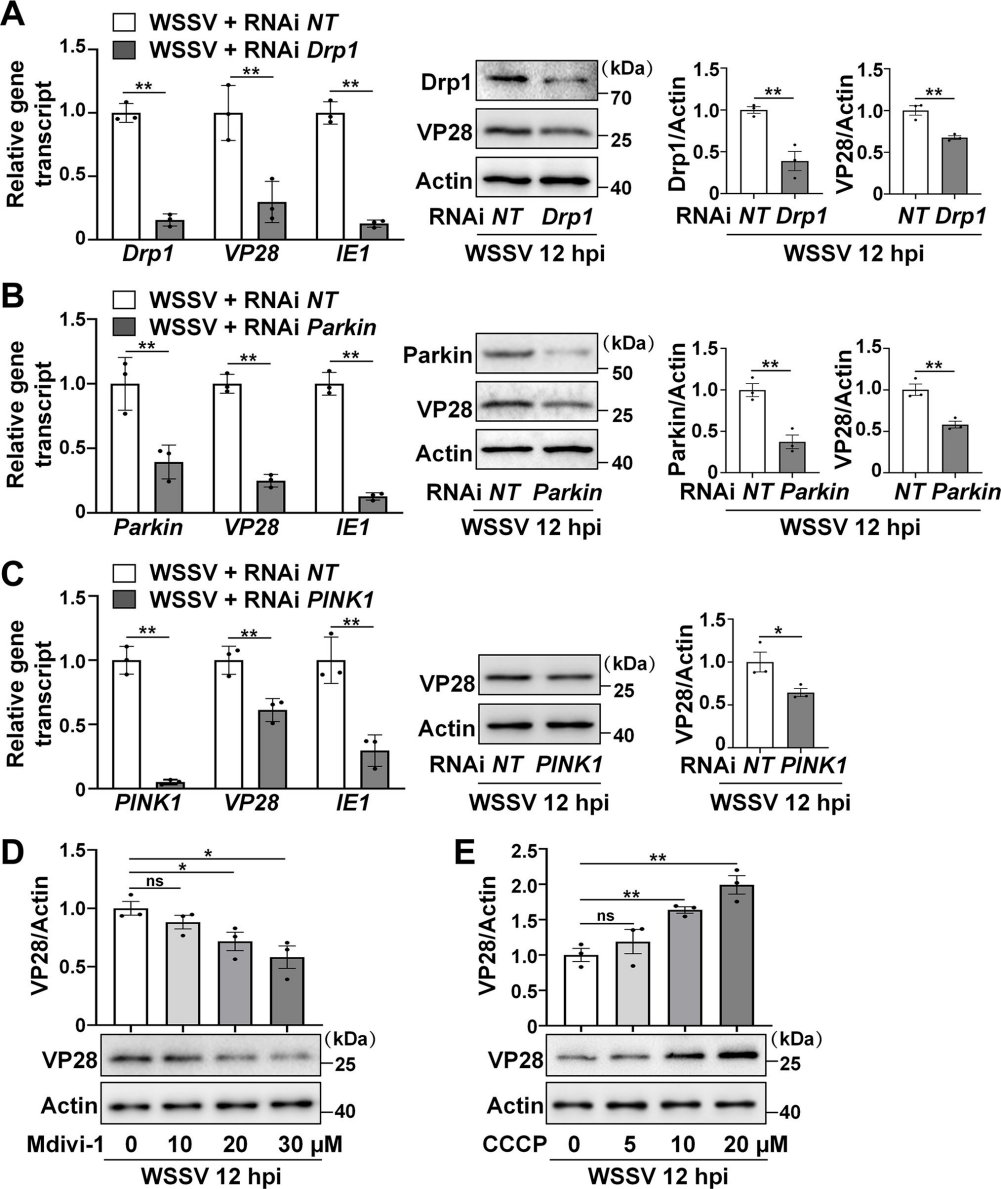
Incomplete mitophagy induced by WSSV promotes viral infection. (**A–C**) Disruption of mitophagy reduced WSSV infection. Key mitophagy-related genes, *CqDrp1* (A, left panel), *CqParkin* (B, left panel), and *CqPINK1* (C, left panel), were inhibited by gene silencing, leading to a significant decrease in both viral gene transcription (left panels) and viral protein expression (middle panels). Statistical analyses of the corresponding protein ratios are shown in the right panels. (**D**) Inhibition of *Cq*Drp1 suppressed WSSV infection. Pharmacological inhibition of *Cq*Drp1 with Mdivi-1 reduced WSSV infection in Hpt cells in a dose-dependent manner, as indicated by decreased expression of the viral protein VP28. (**E**) Induction of mitophagy enhanced WSSV infection. Treatment with the mitophagy inducer CCCP increased viral VP28 protein expression in a dose-dependent manner. All statistical analyses were performed based on at least three independent experiments. Significant differences were analyzed using Student’s *t* test: **, *P* < 0.01; *, *P* < 0.05; ns, no significant difference.

To investigate how WSSV-induced incomplete mitophagy influences viral infection, we measured viral gene expression and protein synthesis in Hpt cells under conditions of modulated mitophagy activity. Silencing of genes required for distinct steps of mitophagy, including *CqDrp1* (necessary for mitochondrial aggregation), *CqParkin* (recruited for autophagic degradation of damaged mitochondria), or *CqPINK1* (a key mediator of mitophagy), significantly suppressed the transcription of WSSV genes such as *IE1* and *VP28* at 12 hpi. Consistently, synthesis of the viral structural protein VP28 was also markedly reduced (Fig. 9A through C). Similarly, pretreatment of WSSV-infected Hpt cells with the Drp1 inhibitor Mdivi-1 decreased VP28 protein levels in a dose-dependent manner at 12 hpi (Fig. 9D). Conversely, activation of mitophagy with the inducer CCCP enhanced viral infection, as shown by a dose-dependent increase in VP28 levels (Fig. 9E). Together, these findings indicate that WSSV-induced mitochondrial aggregation is associated with an incomplete mitophagic response, which, in turn, promotes viral infection.

### Wsv156 facilitates WSSV infection by promoting glycolytic reprogramming

**Fig 10:**
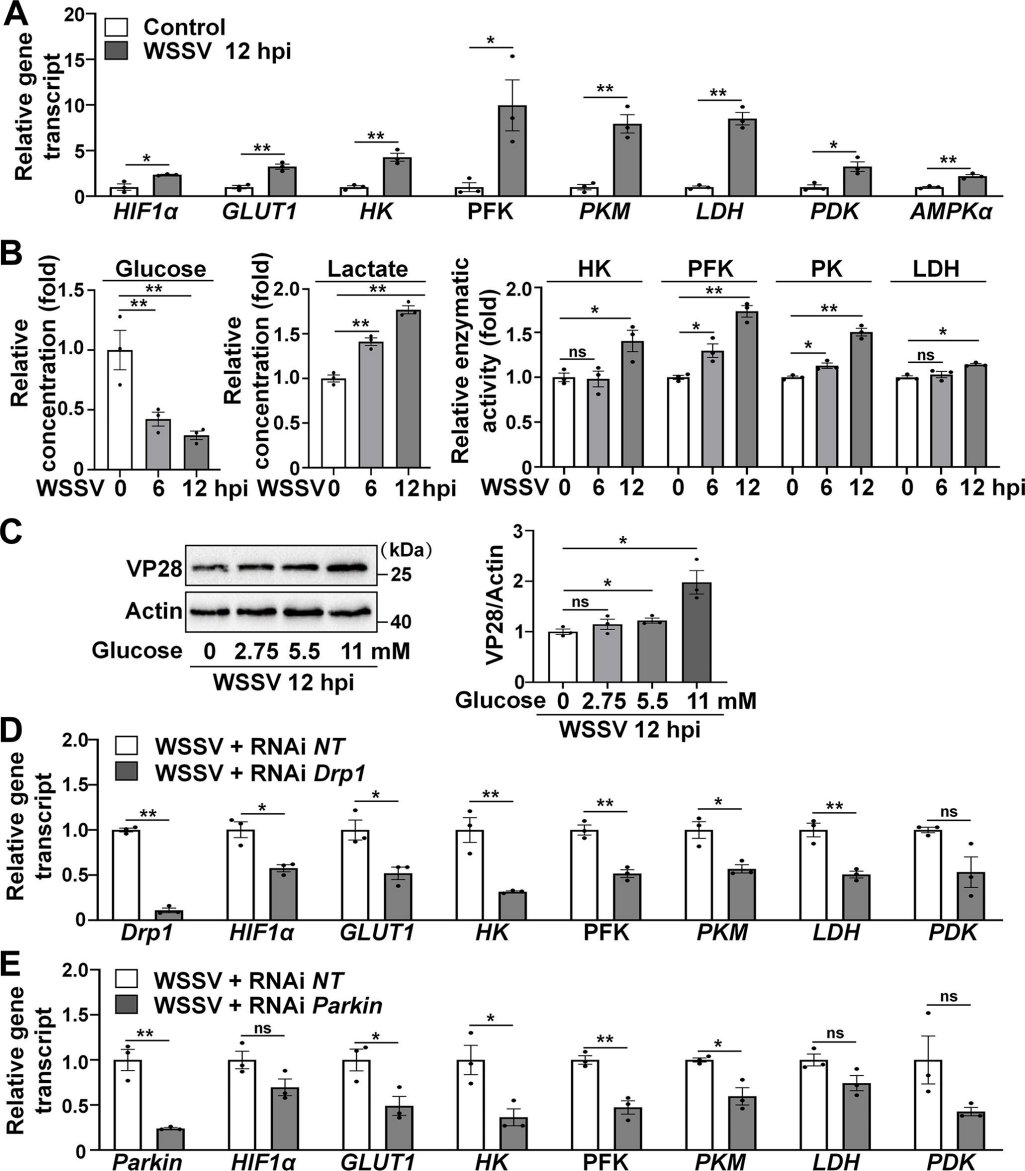
WSSV infection promotes glycolysis via *Cq*Parkin-mediated mitophagy. (**A**) WSSV infection upregulated glycolysis-related gene expression. In crayfish at 12 hpi, key glycolytic genes, including *CqHIF1α*, *CqGLUT1*, *CqHK*, *CqPFK*, *CqPKM*, *CqLDH*, *CqPDK*, and *CqAMPKα*, were significantly upregulated. (**B**) WSSV infection enhanced glycolytic activity in crayfish *in vivo*. WSSV infection increased glucose consumption (left panel), lactate production (middle panel), and the activities of major glycolytic enzymes (HK, PFK, PK, and LDH; right panel) in crayfish. (**C**) WSSV infection was glucose-dependent. VP28 protein expression increased progressively with rising glucose concentration. (**D**) Gene silencing of *CqDrp1* suppressed glycolysis-related gene expression in WSSV-infected cells. (**E**) Gene silencing of *CqParkin* inhibited glycolysis-related gene expression in WSSV-infected cells. All statistical analyses were performed based on at least three independent experiments. Significant differences were analyzed using Student’s *t* test: **, *P* < 0.01; *, *P* < 0.05; ns, no significant difference.

Mitochondria serve as the primary site of ATP production and are central to cellular energy metabolism; mitochondrial dysfunction can thus drive metabolic reprogramming (25). In addition to its interaction with TOMM70, mitochondrial interactome analysis showed that wsv156 interacted with core components of the electron transport chain, including NDUFA9 (Complex I), CYC1 (Complex III), and COX5B (Complex IV), as well as the ATP synthase subunit ATP5F1A (Fig. 2C). This direct multi-target binding likely underlies the observed mitochondrial dysfunction and mito-aggresome formation, which, in turn, may contribute to Parkin-dependent mitophagosome formation and a switch to glycolysis. In line with this, transcriptional analysis revealed that glycolysis-related genes, including *CqHIF1α*, glucose transporter type 1 (*CqGLUT1*), hexokinase (*CqHK*), phosphofructokinase (*CqPFK*), pyruvate kinase M (*CqPKM*), lactate dehydrogenase (*CqLDH*), pyruvate dehydrogenase kinase (*CqPDK*), and AMP-activated protein kinase α (*CqAMPKα*), were significantly upregulated *in vivo* in crayfish Hpt tissue following WSSV infection (Fig. 10A). We next evaluated glycolytic activity by measuring glucose consumption, lactate production, and key enzyme activities. Glucose levels in WSSV-infected hematopoietic tissue were markedly lower than in controls at 6 and 12 hpi (Fig. 10B, left panel), indicating enhanced glucose uptake. Concurrently, lactate production was elevated (Fig. 10B, middle panel), suggesting that the increased glucose flux was directed toward glycolysis. Moreover, activities of key glycolytic enzymes, including the regulatory kinases HK, PFK, and PK, as well as LDH, which catalyzes the final step of reducing pyruvate to lactate, were significantly increased after WSSV infection (Fig. 10B, right panel). Consistent with a metabolic shift toward glycolysis, supplementing extra glucose in Hpt cells significantly enhanced WSSV infection (Fig. 10C). Together, these data indicate that WSSV hijacks host metabolism by upregulating glycolysis to support viral replication. Notably, silencing either *CqDrp1* or *CqParkin* in WSSV-infected Hpt cells downregulated the expression of glycolysis-related genes (*CqHIF1α*, *CqGLUT1*, *CqHK*, *CqPFK*, *CqPKM*, *CqLDH*, and *CqPDK*) (Fig. 10D and E). These findings indicate that WSSV-induced incomplete mitophagy, which relies on *Cq*Drp1 and *Cq*Parkin, contributes to the viral enhancement of glycolysis.

**Fig 11:**
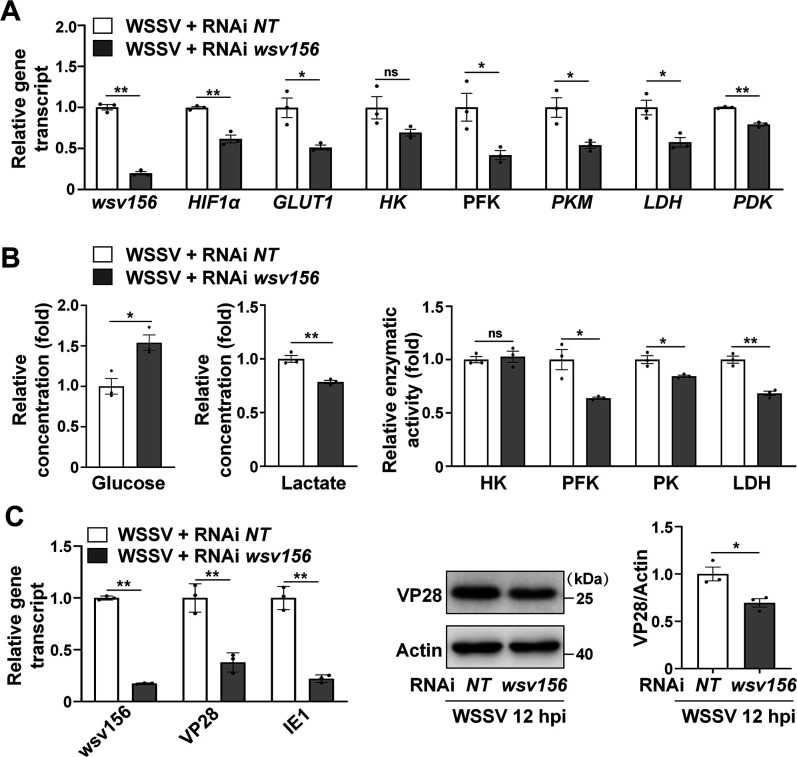
Wsv156 promotes WSSV infection by driving glycolytic reprogramming. (**A**) *Wsv156* silencing downregulated glycolysis-related gene expression in WSSV-infected cells. At 12 hpi, knockdown of *wsv156* in Hpt cells significantly suppressed the expression of key glycolytic genes, including *CqHIF1α*, *CqGLUT1*, *CqPFK*, *CqPKM*, *CqLDH*, and *CqPDK*. (**B**) Wsv156 was required for WSSV-induced glycolysis. *In vivo* knockdown of *wsv156* in WSSV-infected crayfish significantly reduced glucose consumption (left panel) and lactate production (middle panel), and decreased the activity of key glycolytic enzymes (PFK, PK, and LDH; right panel). (**C**) Knockdown of *wsv156* suppressed WSSV infection. Silencing of *wsv156* reduced both viral gene transcription (*IE1* and *VP28*; left panel) and viral protein expression (VP28; middle panel). The expression of VP28 protein was statistically analyzed and is shown in the right panel. All statistical analyses were performed based on at least three independent experiments. Significant differences were analyzed using Student’s *t* test: **, *P* < 0.01; *, *P* < 0.05; ns, no significant difference.

Given that WSSV-induced incomplete mitophagy promotes glycolysis, we next asked whether wsv156, the viral protein responsible for orchestrating this mitophagic response, directly drives metabolic reprogramming. To test this, we assessed the effect of *wsv156* knockdown on glycolytic activity. Silencing of *wsv156* in Hpt cells resulted in a broad downregulation of glycolysis-related genes, including *CqHIF1α*, *CqGLUT1*, *CqPFK*, *CqPKM*, *CqLDH*, and *CqPDK* (Fig. 11A). Consistent with this transcriptional repression, *wsv156* knockdown markedly reduced glucose consumption and lactate production (Fig. 11B, left and middle panels), and decreased the activities of key glycolytic enzymes PFK, PK, and LDH in crayfish *in vivo* (Fig. 11B, right panel). Consequently, both viral gene expression and protein synthesis were strongly suppressed (Fig. 11C). Together, these coordinated changes suggest that efficient viral replication depends on wsv156 function and is tightly coupled to an upregulated glycolytic state in host cells.

### Wsv156 promotes WSSV infection by inhibiting apoptosis

**Fig 12:**
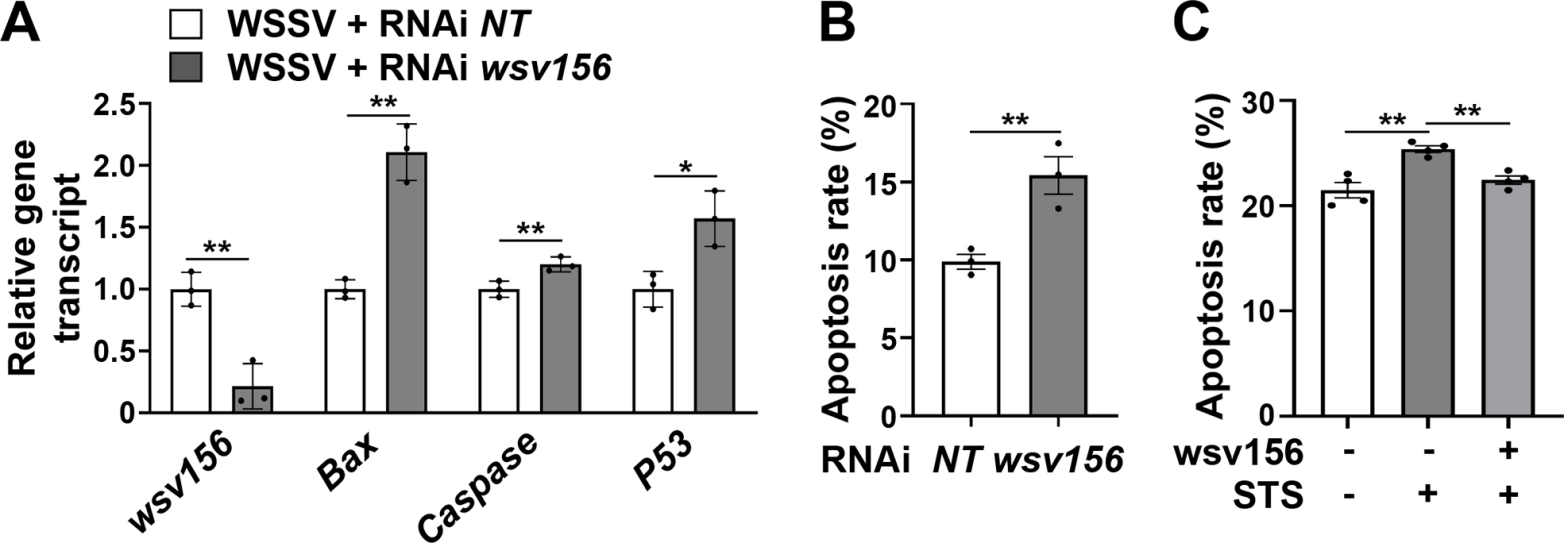
Wsv156 promotes WSSV infection by suppressing apoptosis. (**A**) Silencing of *wsv156* upregulated apoptosis-related gene expression. In Hpt cells at 12 hpi, knockdown of *wsv156* significantly increased transcript levels of the apoptosis-related genes *CqBax*, *CqCaspase*, and *CqP53*. (**B**) *Wsv156* knockdown enhanced apoptosis in WSSV-infected Hpt cells. *Wsv156* gene silencing significantly promoted apoptosis in Hpt cells following WSSV infection, as determined by Annexin V-FITC/PI staining and flow cytometry. (**C**) Overexpression of wsv156 inhibited apoptosis. Overexpression of wsv156 in HEK 293T cells attenuated STS-induced apoptosis, as analyzed by Annexin V-FITC/PI staining and flow cytometry. All statistical analyses were based on at least three independent experiments. **, *P* < 0.01; *, *P* < 0.05.

Having established the mitochondrial localization and metabolic reprogramming activity of wsv156 (Fig. 2 and 11), we further explored its interactions within the organelle. By mitochondrial interactome analysis, we found that wsv156 also interacts with CYC1, a core subunit of respiratory complex III that has been implicated in apoptosis regulation through its role in preserving mitochondrial cytochrome c retention (26). We then speculate that the sequestration of damaged mitochondria within Parkin-labeled mitophagosomes physically compartmentalizes pro-apoptotic factors like cytochrome c, thereby suppressing mitochondria-dependent apoptosis, a process that may facilitate viral persistence. Consistent with this model, knockdown of *wsv156* in Hpt cells significantly upregulated key apoptosis-related genes, including Bcl-2-associated X protein (*CqBax*), caspase (*CqCaspase*), and tumor protein p53 (*CqP53*), and increased the apoptotic rate (Fig. 12A and B), indicating activation of apoptotic pathways. Conversely, overexpression of wsv156 in HEK 293T cells suppressed staurosporine (STS)-induced apoptosis, yielding a lower proportion of apoptotic cells compared to vector-transfected controls (Fig. 12C). Together, these results demonstrate that wsv156 promotes WSSV infection by inhibiting host cell apoptosis. Thus, wsv156 functions as a pivotal viral effector that facilitates infection through a dual mechanism: remodeling host metabolism and, via mitochondrial reorganization, blocking mitochondria-dependent apoptotic signaling.

## DISCUSSION

**Fig 13:**
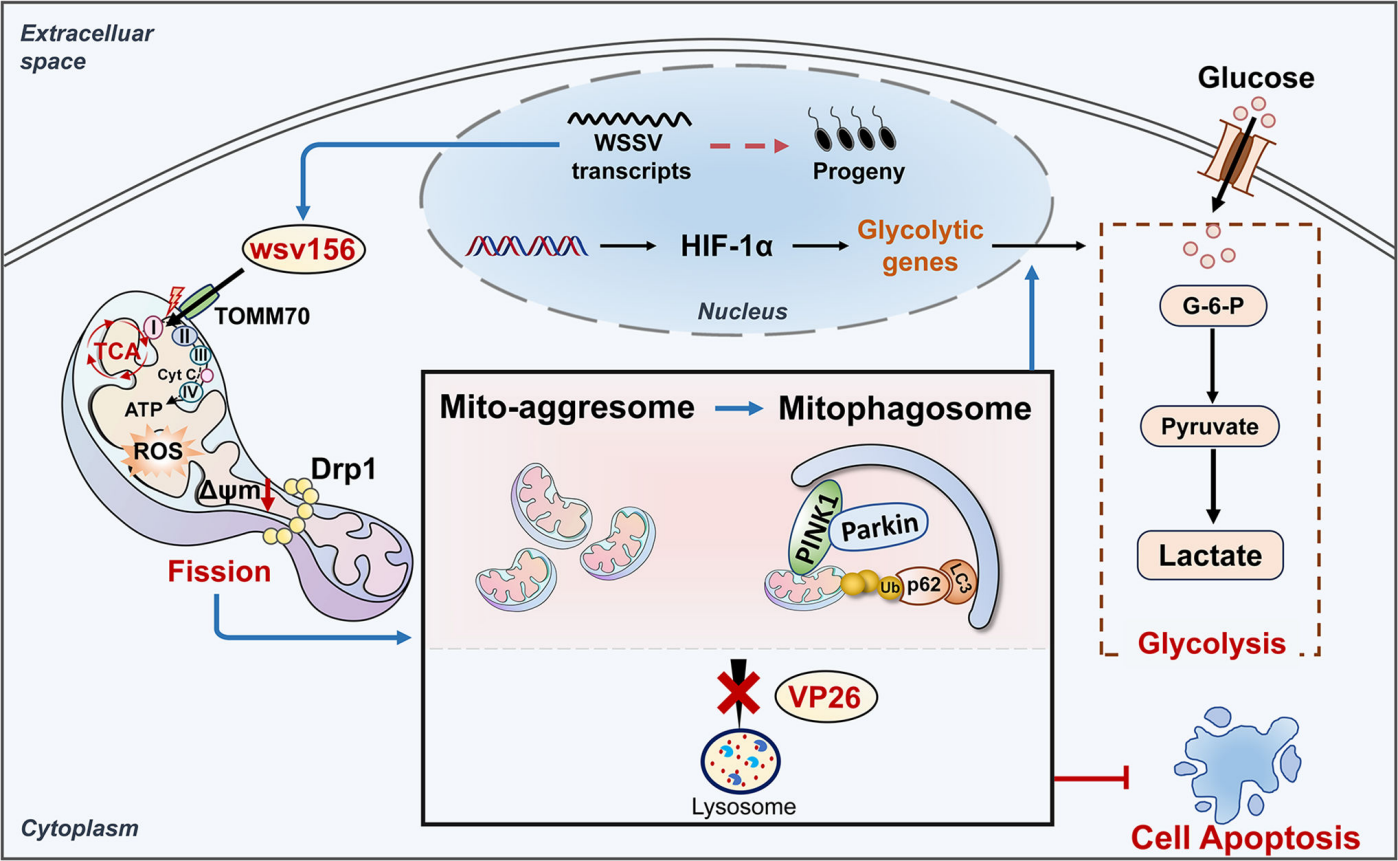
Proposed model: wsv156 facilitates viral infection by hijacking mitophagy. The WSSV-encoded protein wsv156 localizes to host mitochondria and induces mitochondrial dysfunction, recruiting the fission GTPase Drp1 to drive the formation of mito-aggresomes. This aggregated state is associated with activation of the core PINK1–Parkin pathway, initiating mitophagy. However, the viral protein VP26 subsequently blocks mitophagosome-lysosome fusion, resulting in incomplete mitophagy. This arrested process upregulates *HIF1α* expression, thereby promoting glycolysis and inhibiting apoptosis. Collectively, these alterations enhance glycolysis and inhibit apoptosis, thereby creating a cellular environment that facilitates WSSV infection.

Collectively, our findings delineate a sophisticated mechanism by which WSSV hijacks host mitophagy to promote viral replication. Based on our data, we propose the following model (Fig. 13). Upon infection, the viral protein wsv156 targets host mitochondria and recruits *Cq*Drp1, thereby driving mitochondrial fission and aggregation in a Drp1-dependent manner. These mitochondrial aggregates may facilitate the recruitment and activation of the E3 ubiquitin ligase *Cq*Parkin, leading to the formation of *Cq*Parkin-labeled mitophagosomes. However, the viral envelope protein VP26 blocks the subsequent fusion of these mitophagosomes with lysosomes, resulting in incomplete mitophagy. This stalled process supports viral replication through two proposed complementary mechanisms: by upregulating HIF1α expression to enhance glycolysis and by sequestering mitochondria-derived pro-apoptotic factors to suppress host-cell apoptosis.

Growing evidence indicates that numerous viruses manipulate mitochondrial dynamics to promote infection, frequently by targeting Drp1-mediated fission. For instance, viral proteins like HBV X protein (HBx) and hepatitis C virus (HCV) non-structural 5A protein (NS5A) activate Drp1 to induce mitochondrial fragmentation, leading to subsequent Parkin-dependent mitophagy (27, 28). Similarly, NDV and porcine reproductive and respiratory syndrome virus (PRRSV) have also been shown to shift the mitochondrial fusion–fission balance toward fission, thereby facilitating the process of mitophagy (21, 29). In line with this established paradigm of viral-induced mitochondrial fission, our present study reveals that the WSSV protein wsv156 also targets mitochondrial dynamics by recruiting Drp1. Importantly, however, the functional impact of wsv156 extends well beyond the simple induction of fission, exhibiting several distinctive features that are detailed in the following sections. Notably, beyond its role in initiating mitochondrial fission via *Cq*Drp1 recruitment, wsv156 drives the clustering of fragmented mitochondria into prominent aggregates, termed “mito-aggresomes.” This aggregation likely involves mechanisms beyond fission alone, such as inter-mitochondrial tethering or microtubule-mediated transport (30). We observed that the formation of mito-aggresomes is associated with Parkin-dependent mitophagy. A shared functional parallel is observed with the EBV protein BHRF1, which similarly triggers fission and promotes the formation of mito-aggresomes destined for mitophagic degradation (17, 31). Taken together, the formation of mito-aggresomes represents both a conserved viral strategy and a WSSV-specific adaptation. First, by concentrating damaged mitochondria and co-localizing the PINK1–Parkin machinery, mito-aggresomes serve as a signal-amplifying platform, a shared tactic employed by both WSSV and EBV to potentiate mitophagy initiation. Second, and more distinctively, this clustered configuration may function as a sustained “signaling depot” that drives mitophagosome formation even in the absence of lysosomal clearance, a state we propose contributes to enhanced glycolysis and ultimately supports WSSV propagation.

Furthermore, our mass spectrometry analysis provides crucial molecular insights into how wsv156 achieves such comprehensive mitochondrial perturbation. In addition to its previously identified interaction with the outer-membrane translocase *Cq*TOMM70, wsv156 was found to interact with a spectrum of core mitochondrial proteins. These include key components of the electron transport chain (e.g., NDUFA9 of Complex I, CYC1 of Complex III, COX5B of Complex IV), ATP synthase (ATP5F1A), and metabolic enzymes such as HADHA involved in fatty acid β-oxidation. This multi-target-binding profile distinguishes wsv156 from other viral proteins that perturb mitochondria indirectly, a strategy exemplified by PRRSV and porcine circovirus 2 (PCV2), which act primarily through inducing oxidative stress or calcium dysregulation (29, 32). The direct interaction of wsv156 with respiratory chain components strongly suggests a direct impairment of oxidative phosphorylation (OXPHOS), which would be expected to compromise mitochondrial metabolic capacity and potentially elevate ROS production. Supporting this interpretation, independent metabolic profiling studies have demonstrated that WSSV infection induces a pronounced shift toward aerobic glycolysis, glutaminolysis, and *de novo* nucleotide synthesis (7). Our findings position wsv156-induced mitochondrial damage as a proximal trigger upstream of these metabolic alterations, thereby providing a direct mechanistic link to the “Warburg-like effect” observed in WSSV-infected shrimp.

Numerous viruses exploit the highly conserved PINK1–Parkin pathway, a central ubiquitin-dependent mitophagy mechanism, to reshape host cell physiology. Consistent with this broader pattern, our data demonstrate that wsv156-induced mito-aggresomes robustly activate this canonical cascade in crustaceans. Crucially, knockdown of *Cq*Parkin markedly suppressed the formation of mitophagosomes, establishing that this E3 ubiquitin ligase is essential for WSSV-induced mitophagy. The reliance of WSSV on the PINK1–Parkin pathway parallels its recruitment by other viruses. For instance, VZV glycoprotein E and EBV BHRF1 each promote Parkin-dependent mitophagy to dampen type I interferon (IFN) production (15, 17). Similarly, HBV and HCV exploit Drp1- and Parkin-mediated mitophagy to sequester or eliminate mitochondria harboring pro-apoptotic signals, thereby attenuating apoptosis and facilitating persistent infection (27, 33). Further examples include PRRSV and classical swine fever virus (CSFV), which trigger PINK1–Parkin-dependent mitophagy to drive a glycolytic shift that supports viral replication (29, 34). Collectively, although the functional outcomes vary, encompassing immune evasion, anti-apoptosis, and metabolic reprogramming, the PINK1–Parkin pathway is a recurrent target for diverse viruses across hosts. It is noteworthy that mitophagy initiation is not universally Parkin-dependent; SARS-CoV-2 (via the receptor NIX) and IAV (via the adaptor TOLLIP) engage Parkin-independent routes (16, 35). The observation that WSSV, despite the availability of such alternative mechanisms, has specifically evolved to rely on the canonical PINK1–Parkin machinery underscores its functional importance as a conserved target for viral manipulation, even in evolutionarily distant hosts. This convergence highlights a fundamental role for the PINK1–Parkin pathway in host–pathogen interactions, extending its relevance to invertebrate–virus systems.

Beyond initiating mitophagy, WSSV actively arrests the process in an incomplete state to facilitate infection, primarily via its envelope protein VP26. We previously reported that VP26 inhibits autophagosome–lysosome fusion by sequestering SNAP29, a core Qb-SNARE essential for autophagosomal maturation (11). Here, we demonstrate that VP26 executes the same blockade specifically on mitophagic flux, thereby maintaining mitophagy in an incomplete state. Knockdown of *VP26* restores mitochondrial degradation, confirming that SNAP29 sequestration is the key mechanism underlying stalled mitophagy in WSSV-infected cells. This “initiate-but-block” strategy mirrors that employed by HPIV3, which uses a dedicated two-protein system: the matrix (M) protein triggers mitophagy, while the phosphoprotein (P) specifically blocks autophagosome–lysosome fusion by similarly binding SNAP29, thereby interfering with RIG-I signaling and potentially generating membranous replication scaffolds (18, 36). Other viruses, such as SARS-CoV-2 (via Nsp8) and human immunodeficiency virus (HIV, via gp120/Tat), have also been reported to induce incomplete mitophagy, albeit through distinct, SNAP29-independent mechanisms (19, 37). The convergent evolution of incomplete mitophagy across phylogenetically diverse viruses, ranging from RNA viruses to large DNA viruses, strongly suggests that this arrested state confers conserved functional advantages. These likely include sustaining mitochondrial damage signals that stabilize HIF1α to promote glycolysis, sequestering pro-apoptotic factors within undegraded mitophagosomes, and avoiding bioenergetic stress from excessive mitochondrial clearance. In some viral systems, arrested autophagic membranes are also proposed to serve as scaffolds for viral assembly or signaling complex formation. WSSV is distinct in that its progeny virions assemble and are enveloped entirely within the host cell nucleus, rather than on cytoplasmic autophagic membranes. Nevertheless, our functional experiments confirm that the incomplete mitophagic state is critical for viral replication. Overall, this “initiate-but-block” strategy represents a sophisticated functional integration of metabolic reprogramming and apoptosis suppression, tailored to support sustained viral proliferation.

The core components of the Parkin-dependent mitophagy pathway, including Drp1, PINK1, Parkin, and GB/LC3, are evolutionarily conserved from crustaceans to mammals. Importantly, the key functional effects of wsv156—mitochondrial aggregation, Parkin recruitment, GB-II/LC3-II lipidation, mitochondrial protein degradation, and anti-apoptotic activity—were consistently observed in both crayfish primary Hpt cells and human HEK 293T cells. This cross-system consistency strongly suggests that wsv156 targets a deeply conserved mitochondrial quality control circuit. Nevertheless, we acknowledge the possibility of species-specific differences in regulatory fine-tuning, accessory factors, or isoform composition. While the use of HEK 293T cells validates the conserved nature of the core machinery, it cannot fully recapitulate crustacean-specific regulatory nuances. Future studies employing crustacean-specific knockout or rescue models will be necessary to address these limitations.

In terms of metabolism, successful viral replication commonly requires reprogramming host metabolism to meet heightened demands for energy and biosynthetic precursors. While WSSV infection is known to promote aerobic glycolysis via the PI3K-Akt-mTOR-HIF1α axis, the upstream trigger initiating this metabolic rewiring has remained unclear (6, 38, 39). Our findings identify mitochondrial damage triggered by the viral protein wsv156 as the critical upstream signal. Consistent with this, WSSV infection led to transcriptional upregulation of multiple glycolysis-related genes, including *CqHIF1α, CqGLUT1, CqHK, CqPFK, CqPKM, CqLDH, CqPDK,* and *CqAMPKα* (Fig. 10A). Parallel metabolic analyses showed increased glucose consumption, elevated lactate production, and enhanced activities of HK, PFK, PK, and LDH (Fig. 10B). Moreover, glucose supplementation promoted viral replication in a dose-dependent manner (Fig. 10C). As previously demonstrated, mass spectrometry-based analysis shows that wsv156 directly targets core mitochondrial components, such as electron transport chain subunits and metabolic enzymes, thereby disrupting mitochondrial integrity and bioenergetics. We propose that such damage initiates mitophagy, and that the viral protein VP26 arrests this process in an incomplete state by blocking autophagosome–lysosome fusion. Importantly, it is this incomplete mitophagy, orchestrated jointly by wsv156 and VP26 rather than the mitochondrial damage alone, that serves as the initiating signal driving the upregulation of glycolysis. We speculate that the incomplete mitophagy process induced by wsv156 and VP26 may generate sustained mitochondrial damage signals, potentially mediated by persistent ROS production or an altered redox state, thereby stabilizing HIF1α expression and enhancing glycolysis. For instance, mitochondrial dysfunction and elevated ROS levels induced by porcine epidemic diarrhea virus (PEDV) infection have been shown to promote HIF1α-mediated glycolysis (40). The stalled state of incomplete mitophagy may lead to continuous release of mitochondrial signals (e.g., NAD^+^ depletion or altered metabolite pools), thereby sustaining HIF1α activation. Similarly, NDV has been reported to stabilize HIF1α and trigger metabolic reprogramming through Parkin-dependent mitophagy (21). These findings further support the notion that incomplete mitophagy may serve as a signaling platform. Our data provide correlative evidence: silencing of *wsv156* prevents mitophagy initiation, leading to reduced HIF1α expression and downregulation of multiple glycolysis-related genes (Fig. 11A). Although these data do not directly prove that mitochondrial aggregates function as a “signaling depot,” they are highly consistent with our proposed model.

Moreover, the broad downregulation of glycolysis-related genes observed upon *wsv156* silencing is unlikely to result from a direct transcriptional function of wsv156 itself. Unlike some WSSV proteins with established roles in transcription, such as the early transcription factor wsv181 (41), wsv156 localizes predominantly to mitochondria and lacks canonical nuclear localization signals or DNA-binding domains. Furthermore, our mass spectrometry and interaction analyses indicate that wsv156 physically interacts with multiple core mitochondrial proteins, including respiratory chain subunits and metabolic enzymes, rather than with transcriptional regulators or chromatin-associated factors. Thus, the transcriptional suppression of glycolysis-related genes following *wsv156* knockdown is most parsimoniously explained as an indirect consequence of disrupted mitochondrial homeostasis and mitophagic flux processes that we have demonstrated to be initiated by wsv156. This distinction underscores that wsv156 functions primarily as a mitochondrial perturbator, whose influence on host transcription is mediated through organelle-derived signaling and metabolic rewiring, rather than through direct nuclear gene regulation.

In addition to upregulating glycolysis, the wsv156- and VP26-mediated incomplete mitophagy strongly suppresses mitochondria-dependent (intrinsic) apoptosis. Mechanistically, we propose a model in which the Parkin-dependent sequestration of damaged mitochondria into undegraded mitophagosomes physically traps pro-apoptotic factors such as cytochrome c and prevents their release into the cytosol. Direct evidence, such as subcellular fractionation of cytochrome c or immunoelectron microscopy, will be required to validate this mechanism. This organelle-sequestration strategy constitutes a distinct addition to the known anti-apoptotic arsenal of WSSV. Notably, WSSV encodes at least two anti-apoptotic proteins that exploit complementary strategies: IE1, which activates the pro-survival PI3K-Akt pathway, and WSSV322, a direct inhibitor of effector caspases (42, 43). We propose that the sequestration mechanism acts upstream, by isolating the mitochondrial source of pro-apoptotic signals. Consequently, it provides a complementary layer of protection that works in concert with the virus’s downstream anti-apoptotic strategies. The broad interactions of wsv156 with core mitochondrial proteins, particularly subunits of the respiratory chain and key metabolic enzymes as identified by mass spectrometry, may explain its efficiency in inducing widespread mitochondrial dysfunction, thereby supplying ample initial substrates for this anti-apoptotic “sequestration” mechanism. Therefore, the wsv156-VP26 module may act upstream by physically isolating the source of apoptotic signals. This framework aligns with strategies used by other viruses, such as HBV and HCV, which also leverage mitophagy to counter apoptosis (27, 33). However, WSSV appears to have evolved a refined variant: it engineers a sustained “mitochondrial sequestration” state by initiating mitophagy with wsv156 while concurrently blocking its completion with VP26. This direct, physical-inhibition model may represent an efficient means of apoptosis evasion, potentially crucial for sustaining WSSV’s prolonged infection cycle.

In vertebrate hosts, diverse viruses induce mitophagy to degrade the mitochondrial antiviral signaling (MAVS) protein, thereby suppressing type I interferon production, a strategy finely tuned to a specific immune adaptor (44). In invertebrates, however, the immunological landscape is notably different. Functional MAVS orthologs have been reported only in certain bivalve mollusks such as *Crassostrea gigas* and *Chlamys farreri* (45, 46). Outside this limited lineage, and particularly in arthropods which serve as the primary hosts for WSSV, neither a canonical MAVS ortholog nor a clear functional analog has been identified, indicating that MAVS-dependent antiviral signaling is not a conserved feature across invertebrates. In this context, our study reveals that WSSV, an enveloped large DNA virus that exclusively infects crustaceans, precisely hijacks the evolutionarily conserved PINK1–Parkin pathway to orchestrate incomplete mitophagy. Since its host lacks MAVS, this viral strategy is unlikely to function primarily as a means of evading MAVS-dependent immunity. Instead, our findings demonstrate that WSSV-induced incomplete mitophagy serves principally to drive glycolytic metabolism and suppress apoptosis functions that are experimentally linked to viral replication in this system. A similar repurposing of mitophagy has been reported in other invertebrate viruses; for example, the structural protein VP4 of *Bombyx mori* cypovirus (BmCPV) induces PINK1–Parkin-mediated mitophagy via interaction with host Tom40 to promote viral replication in the silkworm midgut (47). Thus, while the PINK1–Parkin pathway itself is highly conserved, the functional outcomes of its viral co-option differ markedly between vertebrates and invertebrates. These observations reinforce the concept that mitochondria constitute an ancient cellular signaling and metabolic hub. Viruses target this hub not merely because it hosts lineage-specific immune adaptors such as MAVS, but because its core functions, including metabolic regulation, apoptosis control, mitochondrial dynamics, and stress signaling, are universal and essential for cellular homeostasis. By enforcing incomplete mitophagy, WSSV directly exploits these conserved mitochondrial functions to establish a proviral metabolic state while restraining cell death. This principle helps explain the convergent evolution of mitophagy manipulation across phylogenetically distant viruses that infect both vertebrate and invertebrate hosts.

In conclusion, WSSV orchestrates host mitophagy through a precisely regulated, two-phase mechanism: initiation via wsv156 and arrest via VP26. This “trigger-and-trap” strategy not only provides a replicative advantage by rewiring metabolism and inhibiting apoptosis but also reveals a deep evolutionary conservation in how viruses manipulate mitochondrial quality control. Our findings position mitochondrial homeostasis as a central and conserved battlefield in host–virus conflicts and highlight the PINK1–Parkin pathway as a potential target for broadly acting antiviral strategies.

## MATERIALS AND METHODS

### Animals, cell culture, virus preparation, and infection

Healthy male red claw crayfish were purchased from Yuansentai Agricultural Science and Technology Co., Ltd (Zhangzhou, Fujian, China). Prior to experiments, crayfish were acclimatized in aerated tanks at 26°C for at least 2 weeks. Only intermolt, WSSV-free males were used in this study. Hpt cells were isolated from crayfish and cultured at 20°C as previously described by Söderhäll et al. (48) and Liu et al. (49). The WSSV stock was amplified in the red claw crayfish, and prepared virus was quantified by absolute quantification with PCR (50). For infection in Hpt cells, the indicated multiplicity of infection (the ratio of virus to cell) was used as previously described (51).

### Cell line and transfection

HEK 293T cells were cultured in Dulbecco’s modified Eagle’s medium (DMEM; Servicebio, G4511) supplemented with 10% fetal bovine serum (Vazyme, 7E891G4) and 100 U/mL penicillin-streptomycin (Solarbio, P1400) at 37°C in a 5% CO_2_ atmosphere. Plasmid transfections were performed using Lipo293 Transfection Reagent (Beyotime, C0521) according to the manufacturer’s protocol.

### Reagents and antibodies

Chemical inhibitors, CCCP (HY-100941) and Mdivi-1 (HY-15886), were purchased from MedChem Express. STS (S97290) was purchased from Acmec. Alexa Fluor 488/594/647-conjugated secondary antibodies were obtained from YEASEN. MitoTracker Red (C1049B) and 4′,6-diamidino-2-phenylindole (DAPI; SC0110) were from Beyotime. The antibodies used in this study were as follows: anti-β-actin (Cell Signaling Technology, 3700S), anti-α-Tubulin (Zenbio, 250009), anti-VDAC (Zenbio, 380506), anti-TOMM20 (ABclonal, A19403), anti-COX IV (Zenbio, 200147), anti-HA-tag (Proteintech, 66006-1-IG), anti-Myc-tag (Proteintech, 67447-1-Ig), anti-Drp1 (ABclonal, A2586), anti-GABARAP (Abcam, ab109364), anti-ubiquitin (Santa Cruz, sc-8017), anti-LC3B (ABclonal, A19665), HRP-conjugated anti-mouse (Thermo Fisher, 7076P2), and anti-rabbit secondary antibody (Thermo Fisher, 7074P2). Mouse anti-VP28 antibody was kindly provided by Prof. Feng Yang (Third Institute of Oceanography, Ministry of Natural Resources, Xiamen, China). Mouse anti-wsv156, anti-*Cq*TOMM20, anti-*Cq*VDAC, and anti-*Cq*Parkin polyclonal antibodies were produced in our laboratory. Rabbit anti-VP26 polyclonal antibody was produced by Beijing Protein Innovation Co., Ltd.

### Measurement of MMP and ROS

Hpt cells were isolated from healthy crayfish and maintained *in vitro* as described above. For infection, cells were incubated with WSSV or mock-infected. At 3, 6, and 12 hpi, cells were gently detached by repeated pipetting in L15 medium; this procedure did not affect cell viability as confirmed by Trypan blue staining (data not shown). Detached cells were collected by centrifugation, resuspended in serum-free L15 medium, and stained with 200 nM TMRM (Invitrogen, V11042) for MMP measurement or with 10 μM DCFH-DA (Beyotime, S0034) for ROS detection. Staining was performed at 26°C for 30 min in the dark. Following incubation, cells were washed once, resuspended in fresh medium, and analyzed by fluorescence microscopy and flow cytometry.

### Drug treatments

For drug treatments, the Drp1 inhibitor Mdivi-1 (30 μM) or the mitophagy inducer CCCP (10 μM) was added to Hpt cells at 3 hpi and maintained throughout the infection period. For glucose supplementation, cells were cultured in medium containing additional glucose (2.75, 5.5, or 11 mM) during the infection period. Equivalent volumes of DMSO were used as vehicle controls in all experiments.

### TEM analysis

Mitochondrial ultrastructure was examined by TEM in Hpt cells at 12 hpi. Cells were fixed with 2.5% glutaraldehyde at 4°C for 3 h, followed by dehydration through an ethanol series. Samples were then embedded in resin, sectioned using a microtome (Leica, Germany), and double-stained with uranyl acetate and lead citrate before observation under a Hitachi HT-7800 TEM (Japan). Mitochondrial length was measured using ImageJ software (NIH, USA). For each group, at least five cells were analyzed, with 20 mitochondria measured per cell.

### Immunofluorescence assay and mito-aggresomes quantification

Cells were cultured on coverslips and processed for indirect immunofluorescence as previously described (52). Images were acquired using an LSM 780 confocal microscope (Carl Zeiss, Germany). Mitochondrial aggregation was quantified using the CI as previously reported (53). Briefly, CI reflects the degree of mitochondrial compaction within a confined cellular area, calculated from mitochondrial perimeter and area using the formula established by Narendra et al. (53). A CI close to 1 indicates maximal compaction, whereas a CI near 0 indicates minimal compaction. Cells with CI > 0.4 were classified as containing mito-aggresomes, a threshold consistent with prior morphometric analyses of mitochondrial clustering (17, 31). The percentage of cells presenting mito-aggresomes was determined by counting at least 30 random cells in each condition from three independent experiments.

### Gene cloning and sequence analysis

The full-length coding sequences of *wsv156* (GenBank: AAL33160.1), *wsv152* (GenBank: AAL33156.1), *CqDrp1*, *CqPINK1*, and *CqParkin* were amplified by PCR and cloned into the pcDNA3.1 vector to generate fusion proteins with C-terminal HA, Myc, or GFP tags, as indicated. A pcDNA3.1 vector expressing EGFP alone was used as a control.

### RNA interference and reverse transcription-quantitative PCR analysis

For *in vitro* gene silencing, dsRNA was transfected into Hpt cell cultures using Cellfectin II reagent, as previously described (11, 51). *CqParkin, CqPINK1,* and *CqDrp1* were silenced by transfection with dsRNA 24 h before WSSV infection, whereas wsv156 dsRNA was transfected at 3 hpi based on preliminary optimization. For *in vivo* silencing of viral genes, crayfish were injected with VP26 dsRNA (30 µg) 3 h before WSSV infection, or with wsv156 dsRNA (30 µg) at 3 hpi; GFP dsRNA served as the non-targeting control in both protocols. Hpt cells or tissues were collected at 12 hpi for analysis. Knockdown efficiency was assessed at the mRNA level by reverse transcription-quantitative PCR (RT-qPCR), with GFP dsRNA as a non-targeting negative control. Total RNA was extracted using a Spin Column Animal Total RNA Purification Kit (Sangon Biotech, B518651) and reverse-transcribed into cDNA with the PrimeScript RT Reagent Kit (TaKaRa, RR037A). RT-qPCR was performed using a SYBR Green Master Mix (Yeasen) on an ABI 7500 system. The relative expression of target genes was calculated using the 2^−ΔΔCt^ method, with *16S* rRNA as the internal control. Primer sequences are listed in Table S1. Detailed procedures were as previously described (54).

### Mitochondrial and cytoplasmic protein extraction

Mitochondrial and cytosolic fractions were isolated using a Cell Mitochondria Isolation Kit (TransGen Biotech, DE401) according to the manufacturer’s instructions. Purity of the fractions was verified by western blot using antibodies against VDAC or TOMM20 (mitochondrial markers) and Tubulin (cytosolic marker). Western blot analysis was performed as previously described (11).

### Recombinant protein purification and pull-down assays

The coding sequence of *wsv156* was cloned into the pET-28a vector for recombinant expression with an N-terminal His-tag. The recombinant His-tagged protein (rHis-wsv156) was expressed in *E. coli* and purified as previously described (52). For pull-down assays, crayfish Hpt cell lysates were prepared by isolation and solubilization. Purified rHis-wsv156 (10 μg) was incubated with the Hpt lysates, and protein complexes were isolated using nickel-affinity resin according to established protocols (52). Bound proteins were eluted and analyzed by liquid chromatography-tandem mass spectrometry (LC-MS/MS) at APTBIO. The resulting peptide spectra were searched against a custom cDNA library of *Cherax quadricarinatus* compiled in our laboratory for protein identification.

### Co-IP assay

HEK 293T cells were co-transfected with plasmids encoding HA-tagged *Cq*TOMM70 and Myc-tagged wsv156. At 48 h post-transfection, cells were lysed in IP-compatible lysis buffer (Beyotime, P0013). Co-IP was performed using anti-Myc magnetic beads (MedChemExpress, HY-K0206) as previously described (52). Immunoprecipitated complexes were eluted and analyzed by western blot with anti-Actin, anti-Myc, or anti-HA antibodies as appropriate.

### Mito-mCherry-GFP reporter assay

To monitor mitophagic flux, we employed the Mito-mCherry-GFP reporter, a validated tool for assessing mitophagy in cultured cells (55). This assay is based on a tandem mCherry-GFP tag anchored to the mitochondrial outer membrane via the transmembrane domain of human mitochondrial fission 1 protein (FIS1; GenBank: NM_016068.3) (amino acids 101–152). In healthy mitochondria, the tandem fluorescent tag emits both red and green signals that fully overlap, resulting in yellow fluorescence. Upon mitophagy, the engulfed mitochondria are delivered to lysosomes, where acid-sensitive GFP fluorescence is quenched, resulting in red-only puncta that represent mitolysosomes (21, 55). HEK 293T cells were co-transfected with the Mito-mCherry-GFP plasmid and other indicated plasmids, cultured on coverslips for 24 h, and processed for immunofluorescence analysis.

### Metabolite profiling

Crayfish were mock-infected or infected with WSSV, and Hpt cells were collected at 6, 12, and 24 hpi. Glucose, lactate, and the activities of LDH, HK, PFK, and PK were measured using commercial kits (Jiancheng Bioengineering Institute, Nanjing, China) according to the manufacturer’s instructions. Absorbance was read on a BioTek microplate reader and normalized to cell number. To analyze metabolic changes induced by wsv156, crayfish were subjected to wsv156 RNAi at 3 hpi, and Hpt cells were collected at 12 hpi. Metabolite levels and enzyme activities were measured as described above.

### Apoptosis assay

Apoptosis was assessed using an Annexin V-FITC Apoptosis Detection Kit (YeaSen, 40302ES60). Cells were collected, resuspended in binding buffer, stained with Annexin V-FITC and propidium iodide (PI) for 10 min in the dark, and analyzed by flow cytometry. A minimum of 1 × 10⁴ cells per sample was analyzed.

### Statistical analysis

Western blot bands and confocal fluorescence signals were quantified using ImageJ software (NIH, USA). All the graphs presented in this study were created in GraphPad Prism version 8.0 (La Jolla, CA, USA). Pairwise comparisons (e.g., mock- vs. WSSV-infected cells at a single time point, or control vs. gene-specific dsRNA treatments) were analyzed using two-tailed Student’s *t*-tests. For multiple comparisons across time points or experimental conditions (e.g., dose-response or combinatorial treatments), one-way ANOVA with Tukey’s post hoc test was employed. All quantitative data represent the mean ± SD (standard deviation) of at least three independent biological replicates. Significance levels are denoted as: ns, not significant; *, *P* < 0.05; **, *P* < 0.01.

## Data Availability

All data supporting the findings of this study are provided within the article and its supplemental material.

## SUPPLEMENTAL MATERIAL

Supplemental material jvi.00418-26-s0001.docx Fig. S1; Table S1.

ASM does not own the copyrights to Supplemental Material that may be linked to, or accessed through, an article. The authors have granted ASM a non-exclusive, world-wide license to publish the Supplemental Material files. Please contact the corresponding author directly for reuse.
