# Supplementary material for: The white spot syndrome virus wsv156 protein hijacks Parkin-dependent mitophagy to promote viral infection
Source: J Virol. 2026 Jul 29;100(8):e00418-26. doi: 10.1128/jvi.00418-26 (PMC13483479; doi:10.1128/jvi.00418-26)
Supplement: Supplementary file 1 [file jvi.00418-26-s0001.docx]

**S1 Fig.** In silico prediction of the mitochondrial targeting sequence (MTS) of wsv156.

(A) JPred4 secondary structure prediction of full‑length wsv156 (residues 1–62). The predicted MTS (residues 1–25) is boxed in red. α‑helices: red cylinders; β‑strands: green arrows; loops: gray lines. JNETCONF shows prediction confidence (0–9, 9 highest). JNETSOL25/5/0 indicate solvent accessibility (B, buried). JNETJURY asterisks mark consensus of all methods. The N‑terminal region (residues 1–24) is predicted as a high‑confidence α‑helix (JNETCONF 8–9).

(B) Circular network of wsv156 residues 1–25. Residues are colored according to their physicochemical properties: basic (R, H, K) in red, acidic (E) in blue, uncharged polar (S, Q, C) in green, and nonpolar (L, I, F, W, A) in yellow. Edge thickness reflects predicted spatial proximity. Basic residues at positions 2, 5, 7, 9, 11, 14, and 20 cluster on one face, while nonpolar residues at positions 3, 8, 12, 13, 15, 16, 19, 21, and 22 cluster on the opposite face—a hallmark of an amphipathic α‑helix. The acidic residues (E) at positions 23–24 mark the C‑terminal boundary of the predicted MTS.

**S1 Table. The primers used in this study**

| **Primers** | **Sequences (5ˊ-3ˊ)** |
| --- | --- |
| **Cloning of genes** | |
| *Cq*Drp1-ORF-F | ATGGAGGCACTCATCCCCATC |
| *Cq*Drp1-ORF-R | CCACATATGGGTTTCTCTTATC |
| *Cq*Parkin-ORF-F | ATGCTTCGATTCTTCATTGAATTA |
| *Cq*Parkin-ORF-R | GCCAAACCAATGAGCTG |
| *Cq*PINK1-ORF-F | TGGAGTGTGAGGGCTGTG |
| *Cq*PINK1-ORF-R | GAAAGCTCTATTCCATTTTATA |
| *Cq*TOMM70-ORF-F | ATGAGATCAATGTGGAGCG |
| *Cq*TOMM70-ORF-R | CAAGGTACCTAAGTTAGGGATATTG |
| wsv156*-*ORF-F | atgaggttgtcacatcaattg |
| wsv156*-*ORF-R | cattagattccgtgttggttttg |
| wsv152*-*ORF-F | atgacaaggctactcttgc |
| wsv152*-*ORF-R | ccactgttcttggcttcac |
| *Cq*TOMM20-ORF-F | ATGCTTTTTGGAATCTGTTTTGA |
| *Cq*TOMM20-ORF-R | CTCCACATCCTCCTCGGTTA |
| *Cq*VDAC-ORF-F | ATGTCCCCTCCTGTTTATGCT |
| *Cq*VDAC-ORF-R | GGCTTCTAGTTCCATGGC |
| FIS1_aa101-152-F | ccccagaacaaccaggc |
| FIS1_aa101-152-R | ttaggatttggacttggaca |
| **RNAi assay** | |
| ds*Cq*Drp1-F | TAATACGACTCACTATAGGGCTGGAGCTGGAGGAATGGG |
| ds*Cq*Drp1-R | TAATACGACTCACTATAGGGCGGTTTGCTAAGGTTGGGT |
| ds*Cq*Parkin-F | TAATACGACTCACTATAGGGCCGTTGTGGTGTTTGAGTGTGT |
| ds*Cq*Parkin-R | TAATACGACTCACTATAGGGCTGTAGGAGTTCGGCATTTAGG |
| ds*Cq*PINK1-F | TAATACGACTCACTATAGGGAGCTGTTGGAGGCTGTACTTCA |
| ds*Cq*PINK1-R | TAATACGACTCACTATAGGGCATCTGTTCTCTGGGTTCTTGA |
| dswsv156-F | TAATACGACTCACTATAGGGatgaggttgtcacatcaatt |
| dswsv156-R | TAATACGACTCACTATAGGGGCACGAGGAAGAAATGTGCT |
| dsVP26-F | TAATACGACTCACTATAGGGTGGCAACCTAACAAACCTGG |
| dsVP26-R | TAATACGACTCACTATAGGGACAGTTGGCCGAACAGTAAT |
| dsGFP-F | TAATACGACTCACTATAGGGCGACGTAAACGGCCACAAGT |
| dsGFP-R | TAATACGACTCACTATAGGGTTCTTGTACAGCTCGTCCATGC |
| **qRT-PCR** | |
| 16S-qRT-F | AATGGTTGGACGAGAAGGAA |
| 16S-qRT-R | CCAACTAAACACCCTGCTGATA |
| IE1-qRT-F | CTGGCACAACAACAGACCCTACC |
| IE1-qRT-R | GGCTAGCGAAGTAAAATATCCCCC |
| VP28-qRT-F | AAACCTCCGCATTCCTGT |
| VP28-qRT-R | GTGCCAACTTCATCCTCATC |
| *Cq*Drp1-qRT-F | TGGCAAGTCAATAAAGAAAG |
| *Cq*Drp1-qRT-R | TCAACTCTGGGAGACAATC |
| *Cq*Parkin-qRT-F | AGGCCAGATCTCTGCTACCT |
| *Cq*Parkin-qRT-R | AACGGGGGACGAAATGTCTG |
| *Cq*PINK1-qRT-F | GCCAGTTAATACTCTGGGCT |
| *Cq*PINK1-qRT-R | CGCATCTCCTCCATTCTTCT |
| wsv156-qRT-F | GGCACATCAGATGGCTCAAGA |
| wsv156-qRT-R | cacattagattccgtgttg |
| VP26-qRT-F | TCAACGACATTACTGTTCGG |
| VP26-qRT-R | TTCTTGATTTCGTCCTTGAT |
| *Cq*HIF1α-qRT-F | GAAGAAGTGCGGGAA |
| *Cq*HIF1α-qRT-R | CAGTGCCACCAACCAAG |
| *Cq*Glut1-qRT-F | GGTCACCGTCGGTCTTCT |
| *Cq*Glut1-qRT-R | AGGGCTCTGCGTGCTTCA |
| *Cq*HK-qRT-F | CGAATTTCTGGCTGACTG |
| *Cq*HK-qRT-R | GGCATCATTGAGCATCTT |
| *Cq*PFK-qRT-F | GCAGGATGTGCCTATCACCG |
| *Cq*PFK-qRT-R | ATGGCTCCGTCTCTGGGGTA |
| *Cq*PKM-qRT-F | GTCATGGTACGAGAAGTGCT |
| *Cq*PKM-qRT-R | GATCAACTGGAACACCTGGC |
| *Cq*LDH-qRT-F | CACTTCTTGGGCTATTGGT |
| *Cq*LDH-qRT-R | TGTGTGAGTGTTTGCTTGA |
| *Cq*PDK-qRT-F | CAATTCTCTTCGGTGATGGT |
| *Cq*PDK-qRT-R | AGGCGTTTTGTCCCCATT |
| *Cq*AMPKα-qRT-F | GTGTAGTGCGTTTACTCTTG |
| *Cq*AMPKα-qRT-R | AATTTCTCACAGACTTCAGC |
| *Cq*Bax-qRT-F | ATTACCAAATCTCACCACCCG |
| *Cq*Bax-qRT-R | TTCAGGCACTTCCAAATGGTT |
| *Cq*caspase-qRT-F | GGGAGCGTAATGGCACTG |
| *Cq*caspase-qRT-R | GTGACCATCTTTACCCCATAGT |
| *Cq*P53-qRT-F | TGTAAATGTGCTGCTGAGAGAGT |
| *Cq*P53-qRT-R | GGTTTCTCCTGGTGGTGGG |
